# Cascade-targeted delivery platform enhances antigen cross-presentation and STING activation for durable cellular immunity

**DOI:** 10.1016/j.bioactmat.2025.08.033

**Published:** 2025-09-03

**Authors:** Yuan Xue, Shuting Bai, Yating Wang, Jiaxing Feng, Kun Xiong, Xue Tang, Chunting He, Yanhua Xu, Hongling Yu, Tianyi Luo, Qing Lin, Xun Sun, Ling Zhang, Zhirong Zhang, Tao Gong

**Affiliations:** a Key Laboratory of Drug Targeting and Drug Delivery Systems of Ministry of Education, West China School of Pharmacy, Sichuan University, Chengdu 610041, China; b Chengdu Origen Biotechnology Co. Ltd, Chengdu, 610036, China; c Department of Anesthesiology, Laboratory of Anesthesia and Critical Care Medicine, National-Local Joint Engineering Research Centre of Translational Medicine of Anesthesiology, West China Hospital, Sichuan University, Chengdu, 610041, China; d College of Polymer Science and Engineering, Sichuan University, Chengdu, 610065, China

**Keywords:** Nano-emulsion, Cascade-targeted delivery, Endoplasmic reticulum targeting, Antigen cross-presentation, STING activation, CD8^+^ T cell response

## Abstract

Achieving robust and durable cellular immunity remains a key challenge in the development of subunit vaccines, primarily due to inefficient antigen cross-presentation and inadequate immune activation. Here, we engineered a series of nano-emulsions by conjugating human serum albumin (HSA) with fatty acids of varying chain lengths. Through systematic screening, the palmitic acid–modified nano-emulsion was identified as the most effective carrier, exhibiting intrinsic self-adjuvant properties and a strong capacity to elicit cellular immune responses. Notably, this formulation enables cascade-targeted delivery, trafficking sequentially from lymph nodes to antigen-presenting cells (APCs), and ultimately to the endoplasmic reticulum (ER). Upon co-delivery of the model antigen ovalbumin (OVA) and a stimulator of interferon genes (STING) agonist, the nano-emulsion facilitates both efficient antigen cross-presentation and precise intracellular activation of the STING pathway. This synergistic mechanism significantly enhances CD8^+^ T cell responses and promotes durable memory formation, resulting in potent antitumor efficacy in murine models. Collectively, this study presents a safe and versatile nano-emulsion platform that overcomes key barriers in subunit vaccine delivery, offering a promising strategy for next-generation vaccine design.

## Highlights

• Palmitic acid–modified albumin nano-emulsion enables cascade-targeted delivery.
• Cascade targeting from lymph nodes to APCs then to ER enhances immune activation.
• ER-targeted co-delivery boosts antigen cross-presentation and STING activation.
• Self-adjuvanting nano-emulsion elicits CD8^+^ T cell and memory responses.
• Vaccines synergizes with anti–PD-1 antibody to enhance tumor suppression and survival.

## Graphical abstract

**Figure:**
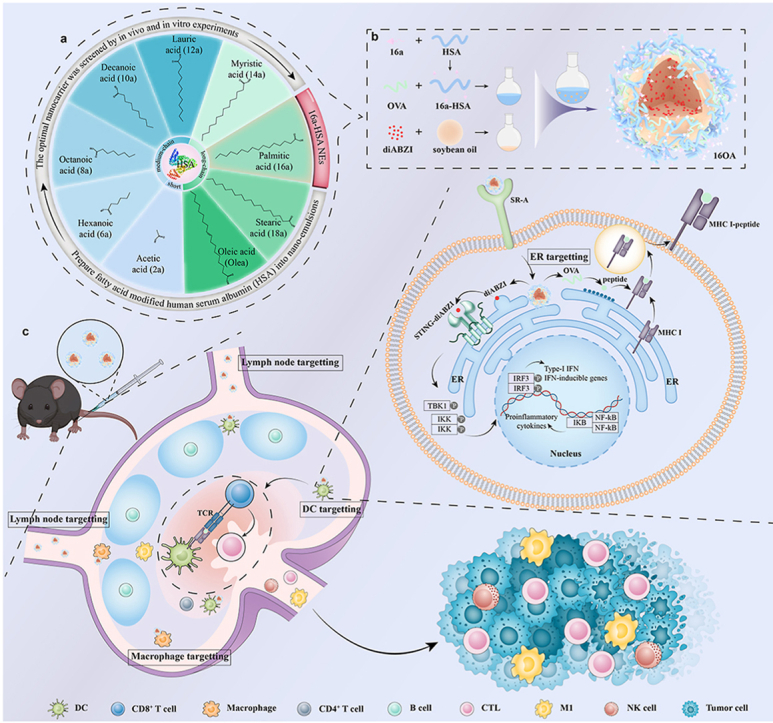

## 1 Introduction

Subunit vaccines, composed of recombinant proteins or synthetic peptides, possesses significant advantages, such as precise immunogen selection, ease of large-scale production, and the safety afforded by controllable ingredients [[1], [2], [3]]. Owing to these benefits, subunit vaccines are anticipated to become widely used worldwide. However, subunit antigens are inherently weak in immunogenicity, necessitating the addition of appropriate adjuvants to elicit a robust immune response [4,5]. Increasing evidence suggests that the co-delivery of antigens and adjuvants is an effective strategy for enhancing subunit vaccine efficacy [6,7], marking a new direction in overcoming this challenge. However, the differing properties of antigens and adjuvants, as well as their potential distinct targets, present a significant hurdle in designing delivery vehicles that can effectively deliver both simultaneously. As a result, existing co-delivery systems have yet to address issues such as weak cellular immune responses and short durations of protection [8,9]. Therefore, the development of a safe and efficient targeted delivery system for subunit vaccines is urgently needed.

Regulating the type and intensity of the immune response using delivery system has long been a major challenge in vaccine development [10,11]. Generally, antigen-presenting cells (APCs) internalize foreign antigens and present them to CD4^+^ T cells via major histocompatibility complex (MHC) II molecules through the lysosomal pathway, primarily initiating Th2-type and humoral immune responses, while cellular immune responses, dominated by CD8^+^ T cells, often remain weak [[12], [13], [14]]. Therefore, to strengthen cellular immune responses, vaccine carriers must enhance the cross-presentation pathway to load exogenous antigens onto MHC I molecules for presentation to CD8^+^ T cells. [15,16]. The endoplasmic reticulum (ER) is a critical position for MHC I molecule synthesis and the binding of MHC I molecules with epitope peptides [17]. Therefore, directly delivering antigens to the ER holds great promise for enhancing antigen cross-presentation [18,19]. Additionally, the ER is also pivotal for the expression of various sensor [20,21], such as STING, a regulatory protein involved in recognizing danger signals from bacteria or viruses and initiates immune response, that is located on the ER membrane [[22], [23], [24]]. Activating the STING pathway in dendritic cells (DCs) and macrophages can effectively enhance APC activation and increase the intensity of the immune response [25]. Conversely, direct activation of STING in T cells could inhibit T cell proliferation and induce apoptosis [[26], [27], [28]]. Moreover, STING activation can also induce apoptosis in B cells [29,30],or elicit regulatory B cells compromising NK function [31]. Obviously, cell-specific STING activation is more appropriate for vaccine adjuvant. Although various delivery systems for STING agonists have been developed, achieving tissue- and cell-specific delivery remains a significant challenge [25,32]. Furthermore, STING agonists often fail to reach the ER to activate STING proteins after entering cells, as most are at risk of lysosomal degradation [32,33]. Therefore, the co-delivery of antigens and STING agonists to the ER of APCs can simultaneously enhance antigen cross-presentation and specific activation of the STING signaling pathway, thereby achieving CD8^+^T cell activation while augmenting immune response. However, most current vaccine delivery studies focus only on organ or cellular levels, highlighting a gap in precise intracellular organelle targeting.

Fatty acids (FAs) are not only a source of energy in the body but also a critical component of its internal structure [34,35]. Their role in effecting immune response has gradually been investigated and elucidated [36,37]. Studies have shown that various characteristics of FAs, such as chain length and degree of saturation, play distinct roles in immune cells, contributing to either pro-inflammatory or anti-inflammatory responses [[38], [39], [40], [41], [42], [43], [44], [45]]. Interestingly, our previous studies have also demonstrated that certain fatty acid-modified albumin nanocarriers can target scavenger receptors and be actively transported into macrophages, thereby facilitating the treatment of related diseases [46,47]. To date, despite individual promising results from studies using FA mimetics or selected natural FAs in vaccine delivery [48,49], systematic investigations into their structural diversity, immunological mechanisms, and therapeutic efficacy remain limited.

**Fig. 1:**
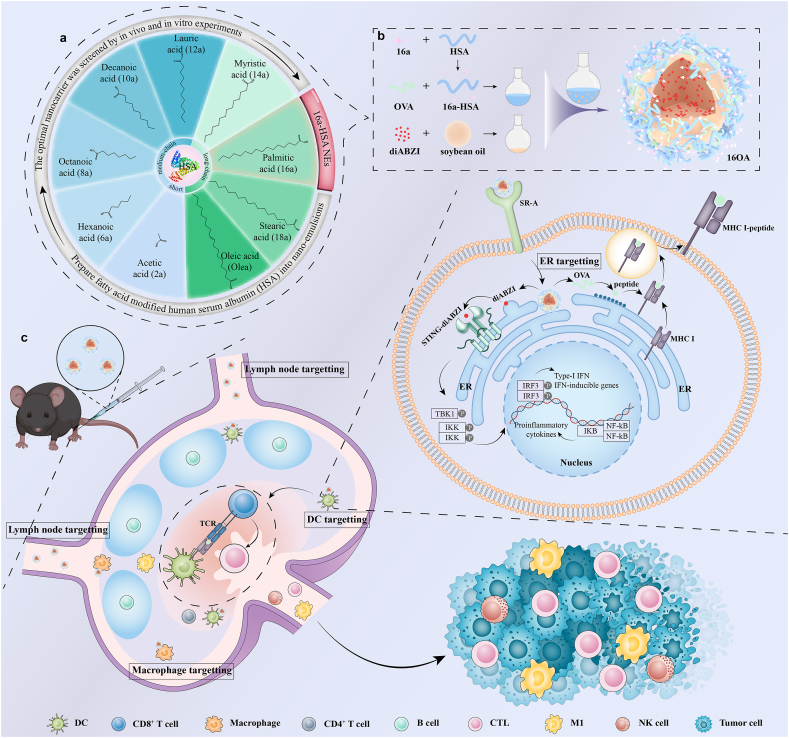
**The palmitic acid-modified HSA nano-emulsions (16a-HSA NEs) served as the efficiently cascade targeted delivery system of subunit vaccine to fight against tumors. a**, We prepared fatty acid-modified human serum albumin (HSA) nano-emulsions (NEs) and identified the optimal subunit vaccine delivery vehicle, palmitic acid-modified HSA NEs (16a-HSA NEs), through both *in vivo* and *in vitro* experiments. **b**, Preparation pathway of 16a-HSA and 16a-HSA NEs (which was loaded with OVA and diABZI, namely 16OA). **c**, After vaccination with 16OA, the NEs exhibited the ability of targeted delivery from secondary lymphoid tissues (lymph nodes) to APCs (DCs and macrophages) and finally to organelles (endoplasmic reticulum, ER, in DCs). Then the co-delivery of antigens (OVA) and STING agonists (diABZI) to the ER simultaneously enhance antigen cross-presentation and specific activation of the STING signaling pathway, thereby maximizing immune response efficiency, especially the cellular immune responses, further realizing the efficient and long-term protection against tumors.

In this study, as depicted in Fig. 1a, a series of FAs (primarily saturated or monounsaturated with varying chain lengths in non-essential FAs) were used to modify HSA, which were then prepared into nano-emulsions (NEs). By comparing the ability of these FA-modified HSA (FA-HSA) NEs to deliver ovalbumin (OVA) and induce immune responses, we found that the palmitic acid-modified HSA (16a-HSA) NEs showed the most potential as a subunit vaccine delivery carrier, not only serving as a delivery vehicle but also functioning as a self-adjuvant. Further research revealed a significant mechanism underlying the phenomenon: the ER-targeting capability of 16a-HSA NEs, which likely enhances antigen cross-presentation and strengthens CD8^+^ T cell responses. Then we prepared 16a-HSA NEs co-delivered the STING agonist (diABZI) with OVA (16OA) (Fig. 1b). Moreover, as exhibited in Fig. 1c, Further research revealed that 16a-HSA NEs could accumulate in lymph nodes and be extensively internalized by DCs and macrophages. Thus, this NEs represented a cascade targeted delivery system, progressing from lymphoid tissues to APCs and finally to organelles. Ultimately, 16a-HSA NEs demonstrated that the precise co-delivery of OVA and diABZI to the ER simultaneously enhance antigen cross-presentation and specific activation of the STING signaling pathway, thereby maximally inducing systemic antigen-specific immune responses, especially the cellular immune responses, further achieving efficient and long-term protection against tumors. In the tumor prevention model involving repeated tumor cell challenges, the 16OA group elicited a robust protective response, resulting in tumor-free survival in 89 % of mice. In the therapeutic tumor model, the combination of 16OA with anti-PD-1 antibody yielded a notable outcome, with 62.5 % of tumor-bearing mice exhibiting complete tumor clearance and sustained survival.

## 2 Results and discussion

### 2.1 Palmitic acid modification stood out among a series of fatty acid modifications

We modified HSA using a series of saturated or unsaturated FAs with varying carbon chain lengths, including acetic acid (2a), hexanoic acid (6a), octanoic acid (8a), decanoic acid (10a), lauric acid (12a), myristic acid (14a), palmitic acid (16a), stearic acid (18a), as well as the unsaturated fatty acid oleic acid (Olea). The fluorescence emission spectrum (Supplementary Fig. 1) shows that, the maximum emission wavelength of FA-HSA exhibited a blue shift of 14–17 nm compared with HSA, except in the case of 2a-modified HSA (2a-HSA), indicating an interaction between FA (with carbon chain lengths of 6–18) and HSA. Subsequently, compared to HSA, we found that the isoelectric points of FA-HSA were reduced, their degrees of amino substitution were relatively consistent, ranging from 12 % to 20 %, and the molecular weights increased from 66.40 kDa to 66.71–69.59 kDa (Supplementary Table 1). To assess structural changes upon FAs modification, circular dichroism (CD) spectroscopy was conducted. As shown in Supplementary Fig. 2, all FA–HSA conjugates retained the characteristic α-helical signals at ∼208 and 222 nm, though a moderate reduction in α-helical content was observed compared to native HSA. This decrease likely results from lysine modification affecting local protein folding, indirectly confirming successful conjugation. Additionally, the matrix-assisted laser desorption/ionization-time of flight mass spectrometry (MALDI-TOF MS) analysis of HSA and 16a-HSA showed the molecular weight of 16a-HSA increased and the amino substitution degree was 17 % (Supplementary Fig. 3), which aligned with that determined by the ninhydrin method.

The results (Supplementary Table 2) demonstrated that we successfully prepared a series of NEs loaded with OVA (HSA NEs denoted as 0O, and FA-HSA NEs denoted as 2O, 6O, 8O, 10O, 12O, 14O, 16O, 18O, and OleO) using the solvent evaporation method, with particle sizes ranging from 90 to 160 nm, Zeta potentials ranging from −30 to −17 mV, and their encapsulation efficiency (EE) of OVA was greater than 95 %. As seen in Supplementary Fig. 4, these findings revealed that the NEs exhibited excellent storage and serum stability.

**Fig. 2:**
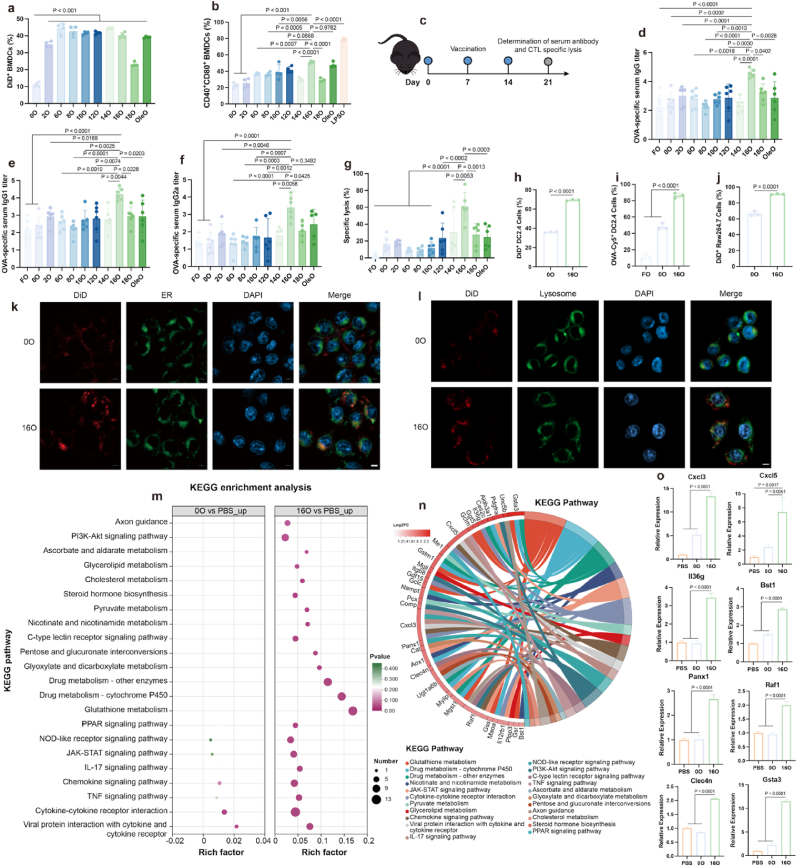
**Screening of subunit vaccine delivery vectors, 16a-HSA NEs stood out among a series of fatty acid-modified HSA NEs. a**, The uptake of DiD load NEs in BMDCs (n = 4 per group). **b**, The expression of costimulatory molecules on BMDCs promoted by FA-HSA and HSA NEs (n = 4 per group). **c**, Schematic representation of the evaluation to investigate the initial induction of specific immune responses in mice by each preparation. **d**-**f**, The levels of antigen-specific antibodies induced in mice by each administration group (n = 5–6 per group): total antibody IgG (**d**), antibody subtype IgG1 (**e**), antibody subtype IgG2a (**f**). **g**, The cytotoxic lysis rate of antigen-specific CTLs induced in mice by each administration group (n = 6 per group). **h**-**j**, The uptake of FA-HSA and HSA NEs on different cells: The uptake of DiD load NEs in DC2.4 cells (**h**) (n = 3 per group), and Raw264.7 cells (**i**) (n = 3 per group), respectively. The uptake of preparations loaded with OVA-CY5 on DC2.4 cells (**j**) (n = 3 per group). **k**, Colocalization of 0O and 16O with the endoplasmic reticulum in DC2.4 cells, scale bar = 5 μm **l**, Colocalization of 0O and 16O with the lysosome in DC2.4 cells, scale bar = 5 μm.**m**-**o**, Transcriptomic changes of BMDCs treated with different preparations (PBS, 0O, 16O) (n = 3 per group): bubble plot for KEGG enrichment analysis of up-regulated genes in BMDCs treated with 0O and 16O compared with the control group PBS, respectively (**m**); Compared with the control group 0O, the KEGG enrichment analysis string plot (**n**) and Relative expression of some genes in BMDCs treated with different preparations (PBS, 0O, 16O) (**o**).

Next, we evaluated the potential of these NEs as delivery vehicles for antigen. At the cellular level, the uptake of FA-HSA NEs by bone marrow-derived dendritic cells (BMDCs) was significantly higher compared to HSA NEs (Fig. 2a), with 16O and OleO demonstrating the most pronounced ability to promote BMDC maturation (Fig. 2b). At the animal level, mice were treated according to the protocol illustrated in Fig. 2c. The results showed that 16O induced the highest levels of OVA-specific antibodies (Fig. 2d–f) and the most significant cytotoxic T lymphocytes (CTLs)-specific lysis effect (Fig. 2g) in mice. In summary, both *in vitro* and *in vivo* results identified 16a-HSA NEs as the most promising delivery vehicle among FA-HSA NEs for subunit vaccines, eliciting the strongest immune responses—likely attributed to their dual function as both a delivery system and a self-adjuvant (Fig. 2b and 2d-g). Notably, they effectively induced the potent cellular immune response.

### 2.2 Palmitic acid modification held the advantages of endoplasmic reticulum targeting and self-adjuvant effect

Given that palmitic acid modification demonstrated superior performance among these FAs, we further investigated its role in cellular and organelle delivery processes to elucidate the underlying mechanisms. We found that the uptake efficiency of 16O by DC2.4 (Fig. 2h and i) and Raw264.7 (Fig. 2j) cells was significantly higher than that of 0O. The uptake inhibition assay (Supplementary Fig. 5) revealed that 16O primarily entered DC2.4 cells through scavenger receptor A with dextran sulfate sodium (DSS) blocking. Additionally, the blank NEs did not affect DC2.4 viability, even at high concentrations (Supplementary Fig. 6), demonstrating that the vector is safe at the cellular level. Interestingly, as shown in Fig. 2k and Supplementary Fig. 7a, following internalization, 16O exhibited predominant colocalization with the endoplasmic reticulum (ER) and minimal overlap with lysosomes. In contrast, 0O showed extensive colocalization with lysosomes and no detectable association with the ER (Fig. 2l, Supplementary Fig. 7b). These results indicated that 16O is primarily distributed within the ER after cellular uptake. Importantly, this ER localization was not attributable to a higher cellular uptake, but rather suggested an inherent ER-targeting property of 16O, which may contribute to improved cross-presentation of antigens and effectively robust induction of CD8^+^ T cell responses [15,17,18]. To elucidate the ER localization of 16O, cells were pretreated with inhibitors or blocking antibodies against FATP and FABP, which mediate fatty acid trafficking to intracellular organelles including the ER [50,51]. These interventions partially reduced colocalization between 16O and ER, as indicated by fluorescence imaging and decreased Manders’ coefficients (Supplementary Fig. 8).

Besides, to further elucidate whether and how the 16a-HSA NEs vector exerted adjuvant effects after entering antigen-presenting cells, we performed transcriptome sequencing analysis on BMDCs treated with different formulations and focused on the transcription genes that were upregulated in 0O and 16O groups compared to the PBS group. And The Kyoto Encyclopedia of Genes and Genomes (KEGG) enrichment analysis indicated that 16O significantly differed from 0O and PBS in multiple immune and metabolic pathways, including key immune-related pathways such as the C-type lectin receptor, NOD-like receptor, cytokine-c ytokine receptor interaction, viral protein interaction with cytokine and cytokine receptor, IL-17, chemokine, and TNF etc. signaling pathway, as well as certain important metabolism-relevant pathways such as drug metabolism - cytochrome P450 and so on (Fig. 2m). Specifically, cytochrome P450, which is mainly located on the ER membrane and responsible for the metabolism and detoxification of exogenous substances [52], indirectly confirms 16O's specific targeting to the ER. Compared to 0O or PBS, the differential genes of 16O in these pathways were largely consistent (Fig. 2n and Supplementary Fig. 9). Analysis of the gene relative expression levels (Supplementary Fig. 10) further revealed that the expression trends of 0O and PBS were similar, while 16O showed relatively significant upregulation. Notably, the results of Fig. 2o indicated that the upregulation of genes in 16O such as Cxcl3, Cxcl5, Il36g, Bst1, Panx1, Raf1, and Clec4n could enhance the subsequent immune response by promoting chemotaxis, migration, antigen presentation, maturation, and cytokine secretion of DCs, while the upregulation of metabolism-related genes such as Gsta3 suggested that 16O modulated different metabolic pathways due to FA modification. In summary, 16O indeed exhibits self-adjuvant properties by modulating the immune-related pathways mentioned above.

Given the excellent ER-targeting capability of 16O, we further encapsulated the STING agonist diABZI into the oil phase of 16O to enhance its ER-targeted delivery. This strategy aims to improve the binding efficiency of diABZI with STING proteins located on the ER membrane, thereby strengthening STING pathway activation. HSA NEs and 16a-HSA NEs each co-loaded with OVA and diABZI named 0OA and 16OA, respectively. 0OA and 16OA held particle sizes of 96/150 nm and zeta potentials of −23/-12 mV, respectively (Supplementary Table 3). Their encapsulation efficiency (EE) of OVA or diABZI was greater than 95 %. Transmission electron microscopy (TEM) image (Supplementary Fig. 11) revealed that the NEs were spherical, with particle sizes of approximately 50 nm for 0OA and 0O, and around 70 nm for 16OA and 16O. The results differed from those obtained using the laser particle size analyzer, likely due to the distinct measurement principles underlying the two techniques. Moreover, 0OA and 16OA, similar to 0O and 16O, exhibited excellent stability (Supplementary Fig. 12), supporting their suitability for subsequent investigations.

### 2.3 Palmitic acid modification performed well in lymph node

**Fig. 3:**
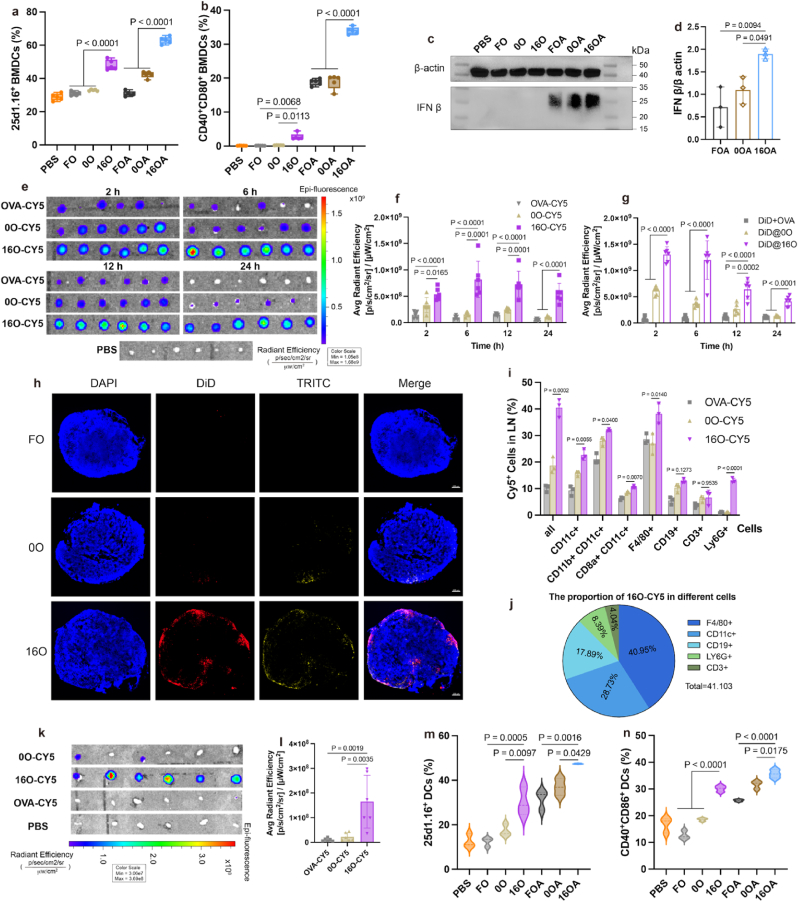
**16a-HSA NEs demonstrated vital advantages as a subunit vaccine delivery vehicle. a-b**, The antigen cross-presentation and maturation induced by different administration groups *in vitro* on DCs: the levels of antigen cross-presentation on DCs *in vitro* (**a**) (n = 5 per group), the levels of maturation on DCs *in vitro* (**b**) (n = 5 per group). **c**, **d**, the levels of the product IFN-β induced by activated STING protein with different administration groups on BMDC: WB images (**c**) and semi-quantitative results (**d**) (n = 3 per group). **e-g**, The fluorescence accumulation in popliteal lymph nodes at 2 h, 6 h, 12 h and 24 h after subcutaneous injection of different preparation groups containing fluorescent dye (n = 6 per group): the fluorescence distribution photographs (**e**) and fluorescence semi-quantitative results (**f**) of lymph nodes after administrating with OVA-CY5 preparations and fluorescence semi-quantitative results of lymph nodes after administrating with DiD preparations (**g**). **h**, The distribution of TRITC and DiD in popliteal lymph node sections at 6 h after subcutaneous injection of preparations co-loaded with OVA-TRITC and DiD, scale bar = 100 μm. **i**, The fluorescence distribution in various cells of the popliteal lymph nodes at 6 h after subcutaneous injection of the preparation loaded with OVA-CY5 (n = 3 per group). **j**, The proportion of various cells in the popliteal lymph nodes of mice with 16O-CY5 uptake at 6 h after subcutaneous injection.**k**, **l**, The fluorescence accumulation in inguinal lymph nodes at 6 h after subcutaneous injection of preparations loaded with OVA-CY5 (n = 6 per group): fluorescence distribution photographs (**k**) and fluorescence semi-quantitative results (**l**). **m-n**, The antigen cross-presentation and maturation induced by different administration groups *in vivo;* the levels of antigen cross-presentation on DCs in lymph node (**m**) (n = 3 per group); the levels of maturation on DCs in lymph node (**n**) (n = 3 per group).

At the cellular level, as illustrated in Fig. 3a, 16O demonstrated the strongest ability to cross-present antigens on BMDCs within the OVA-loaded alone groups, which corroborated the earlier hypothesis regarding the ER targeting of 16O (Fig. 2k) and its ability to induce a robust cellular immune response. Similarly, 16OA was the most prominent in antigen cross-presentation and maturation on BMDCs within the groups co-loaded with OVA and diABZI (Fig. 3a and b). Western blotting (WB) results further supported that 16OA achieved the highest efficiency in the targeting delivery of diABZI for STING activation, resulting in p-TBK1 expression and the greatest production of IFN-β (Fig. 3c–d, Supplementary Fig. 13a). Additionally, the IL-1β, IL-6 and TNF-α secretion levels of 16OA groups were markedly increased (Supplementary Fig. 13b–d), indicating a robust STING pathway activation and BMDCs maturation.

Effective antigen presentation and DC maturation help enhance the activation of specific t cells. Therefore, we also further detected the efficiency of T cell activation *in vitro*. The *in vitro* results indicated that both the 16O and 16OA groups effectively stimulate T cell activation and proliferation in the single-load groups and the co-load groups, respectively (Supplementary Fig14 a-c). And 16OA triggered Granzyme B secretion, presenting a potent cytotoxicity effect (Supplementary Fig14 d). These findings confirmed the strong immunostimulatory capability of our nano-emulsion formulations.

To evaluate the lymph node-targeting capability of the nano-emulsions, CY5-OVA–loaded formulations (OVA-CY5, 0O-CY5 and 16O-CY5) and DiD-loaded formulations (DiD + OVA, DiD@0O, DiD@16O) were subcutaneously injected into mice separately. At various time points, the proximal lymph nodes were collected and imaged using an *in vivo* imaging system to quantify the fluorescence intensity of Cy5-OVA(Fig. 3e–f) or DiD (Supplementary Fig.15 and Fig.3g). The results showed that16O exhibited the highest accumulation in popliteal lymph nodes from 2 to 24 h post-administration compared to other treatment groups (Fig. 3e–g and Supplementary Fig. 15). At 6 h after administration, we examined the distribution of the preparation in sections of the popliteal lymph node. The results revealed that 16O accumulated more effectively within the lymph nodes compared to other treatment groups. (Fig. 3h and Supplementary Fig. 16). Additionally, *in vivo* uptake analysis (Fig. 3i) indicated that 16O had the highest uptake rate among all formulations in total lymph node cells, particularly in DCs, macrophages, and neutrophils. Notably, 16O exhibited superior uptake by both type 1 conventional dendritic cells (cDC1) and type 2 conventional dendritic cells (cDC2) compared to other administration groups. Given that cDC1 is proficient in antigen cross-presentation and activation of CD8^+^ T cells, while cDC2 primarily activates CD4^+^ T cells, and under certain conditions, can also tirgger CD8^+^ T cell responses [53,54], these findings highlighted the considerable potential of 16O to efficiently initiate robust T cell immune responses via cDCs engagement. As shown in Figs. 3j and 16O is primarily taken up by DCs and macrophages in the lymph nodes, highlighting its cell specificity as a delivery carrier. To assess the drainage of nano-emulsions to distal lymph nodes, the fluorescence intensity of Cy5-OVA in inguinal lymph nodes was measured 6 h after administration of Cy5-OVA–loaded formulations. The results showed that at 6 h post-administration, 16O displayed apparent accumulation in the inguinal lymph nodes (Fig. 3k and l), with almost no fluorescence in other administration groups. This observation suggested that 16O also had the ability to target more distant lymph nodes.

Given the superior lymph node-targeting capability of 16O *in vivo*, we further investigated its ability to activate immune responses within lymph nodes. Mice were sacrificed 60 h after subcutaneous administration of each formulation, and popliteal lymph nodes were harvested to prepare single-cell suspensions for flow cytometric analysis. Within the groups loaded with OVA alone, 16O displayed the greatest potential for lymph node activation, demonstrating the strongest capacity to promote antigen cross-presentation and maturation in DCs (Fig. 3m and n). In the groups co-loaded with OVA and diABZI, 16OA similarly exhibited superior performance. Additionally, the STING pathway activation in lymph node was also detected by WB and ELISA. The phosphorylation of TBK1, a key protein in the STING signaling pathway, as well as the secretion of downstream cytokines IFN-α and IFN-β, were also increased in the lymph nodes of the 16OA-treated group (Supplementary Fig. 17). These results collectively indicate that 16a-HSA NEs hold considerable promise as delivery vectors for subunit vaccines.

### 2.4 Palmitic acid modification efficiently triggered antigen-specific immune responses *in vivo*

**Fig. 4:**
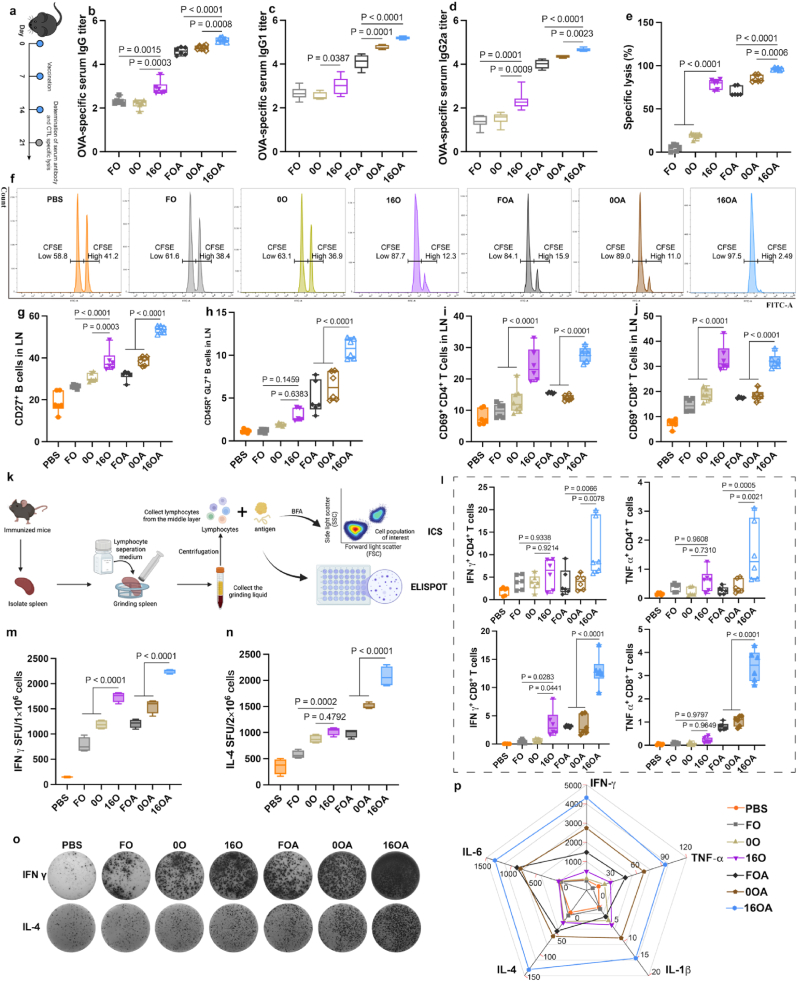
**16a-HSA NEs Efficiently Induce Antigen-Specific Immune Responses *In Vivo*. a**, Evaluation schematic illustration of antigen-specific immune responses in mice following immunization with PBS, FO, 0O, 16O, FOA, 0OA, and 16OA formulations. **b**-**d**, Antigen-specific antibody levels induced by each immunization group in mice (n = 6 per group): total antibody IgG (**b**), antibody subtype IgG1 (**c**), antibody subtype IgG2a (**d**). **e**, **f**, Antigen-specific CTL lysis induced by each immunization group in mice (**e**) (n = 6 per group) and representative flow cytometry analysis plots (**f**). **g**-**j**, Activation of B and T cells within the lymph nodes specifically induced by each immunization group (n = 6 per group): memory B cells (**g**), germinal center (**h**), activated CD4^+^ T cells (**i**), activated CD8^+^ T cells (**j**). **k**, Splenic lymphocytes from immunized mice were isolated and subjected to intracellular cytokine staining (ICS) and ELISPOT assays as illustrated. Created with BioRender.com. **l**, ICS results in splenic T lymphocytes from immunized mice: the level of CD4^+^ or CD8^+^ T cells expressing IFN-γ, TNF-α (n = 6 per group); **m-o**, ELISPOT analysis of IFN-γ (**m**) and IL-4 (**n**) secretion by splenic lymphocytes (n = 4 per group), along with representative images (**o**); **p**, spleen lymphocyte secretion levels of IL-1β, IL-4, IL-6, IFN-γ, TNF-α (pg/mL).

Palmitic acid-modified nano-emulsions demonstrated significant advantages in both antigen presentation and lymph node targeting. Therefore, we further evaluated their ability to elicit specific immune responses *in vivo* in mice (Fig. 4a). The serum of mice immunized was analyzed for anti-OVA antibody levels (Fig. 4b–d). In the NEs group loaded solely with OVA, 16O elicited the highest IgG titer against OVA *in vivo*, while in the NEs group co-loaded with OVA and diABZI, 16OA also induced the highest IgG titer. The trends of the antibody subtypes IgG1 (reflecting the strength of the humoral immune response) and IgG2a (reflecting the strength of the cellular immune response) closely followed the overall IgG titer trend. Antibody responses not only play a crucial role in combating bacterial and viral infections, but increasing evidence also highlights their important function in antitumor immunity [55,56]. Subsequently, we further explored the cellular immune response in immunized mice by assessing the killing efficiency of CTLs *in vivo* (Fig. 4e and f). The results revealed that, consistent with the antibody levels, 16O induced the highest CTL activity among the single OVA-loaded groups, while 16OA induced the highest CTL activity among the OVA and diABZI co-loaded groups. Further evaluation of additional immune indicators in immunized mice showed that, when inguinal lymph node cells were incubated with antigen ex vivo (Fig. 4g–j), 16O induced the highest levels of memory B cells and the most robust activation of CD4^+^ and CD8^+^ T cells compared to other groups loaded OVA alone. In the co-loaded groups, 16OA demonstrated the highest levels of B and T cell activation-related indicators. Additionally, splenic lymphocytes from immunized mice were co-cultured with antigen and blocking agents to inhibit cytokine secretion (Fig. 4k–l and Supplementary Fig. 18). In this setup, 16OA significantly induced higher levels of IL-2, IL-4, IFN-γ, and TNF-α in CD4^+^ T cells and higher levels of IL-2, IFN-γ, and TNF-α in CD8^+^ T cells. Then, 16O predominantly induced a higher level of IFN-γ in CD8^+^ T cells in the single-loading group. ELISPOT results further corroborated these findings (Fig. 4m–o), showing that 16OA was the most effective in inducing spleen lymphocytes to secrete IFN-γ and IL-4 in the co-loaded group, while 16O was most effective at inducing IFN-γ secretion in the single-loaded group. The analysis of T cell cytokines and ELISPOT results suggested that 16O loaded with antigen primarily enhanced the secretion of IFN-γ from CD8^+^ T cells. Moreover, adding the extra adjuvant and then precise co-delivery it and antigen, 16OA can induce comprehensive cytokine secretion from both CD4^+^ and CD8^+^ T cells. Finally, cytokine measurements in the supernatants of co-cultured spleen lymphocytes and antigen were consistent with the above experimental results (Fig. 4p and Supplementary Fig. 19). The 16OA administration group exhibited the highest concentrations of IL-1β, IL-4, IL-6, IFN-γ, and TNF-α. In conclusion, palmitic acid modification can enhance the delivery efficiency of subunit antigens and STING agonists, inducing a robust humoral and cellular immune response simultaneously. Moreover, the blood cell counts of mice in all immunization groups remained within the normal range (Supplementary Fig. 20), suggesting that the designed delivery platform exhibits favorable biocompatibility and systemic safety *in vivo*.

### 2.5 Palmitic acid modification revealed promising in tumor prevention vaccine

**Fig. 5:**
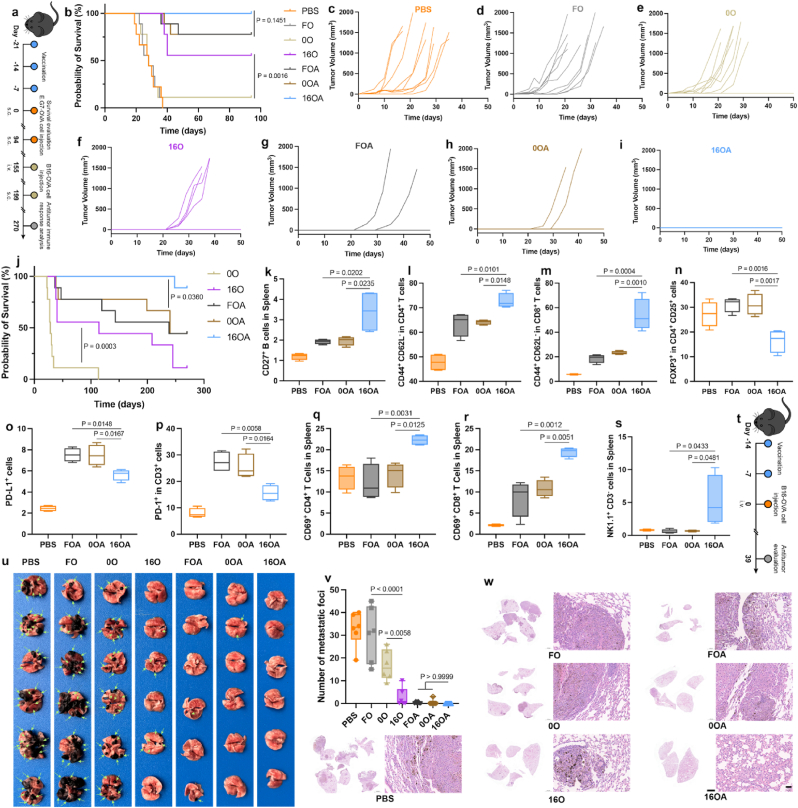
**16a-HSA NEs Effectively Prevent Tumor Development. a**, Schematic of the experimental procedure: mice were immunized with PBS, FO, 0O, 16O, FOA, 0OA, and 16OA vaccine formulations, followed by the first subcutaneous injection of E.G7-OVA tumor cells. Tumor growth and survival were monitored. The remaining surviving mice were subjected to three additional challenges with E.G7-OVA or B16-OVA cells, and the subsequent antitumor immune responses were evaluated. **b**, Survival curves of mice pre-immunized with different formulations following the initial tumor cell challenge. (n = 9 per group). **c**-**i**, Tumor growth curves of mice pre-immunized with PBS (**c**), FO (**d**), 0O (**e**), 16O (**f**), FOA (**g**), 0OA (**h**), and 16OA (**i**) formulations (n = 9 per group). **j**, Survival curves of mice pre-immunized with different formulations following the repeated tumor cell challenges (n = 9 per group). **k**-**s**, Antitumor immune responses in the spleens of mice from each immunization group (n = 4 per group): memory B cells (**k**), CD4^+^ effector memory T cells (**l**), CD8^+^ effector memory T cells (**m**), Treg cells (**n**), all cells expressing PD-L1 (**o**), T cells expressing PD-1 (**p**), activated CD4^+^ T cells (**q**), activated CD8^+^ T cells (**r**), and NK cells (**s**). **t**, Schematic of the experimental procedure for evaluating lung metastasis: mice were immunized with each formulation, followed by tail vein injection of B16-OVA tumor cells. **u**-**w**, Antitumor effects against lung metastasis in pre-immunized mice: images of lung tissues with metastatic foci (**u**) (n = 6 per group), quantification of lung metastatic foci (**v**) (n = 6 per group), and Representative images of lung HE-stained sections, scale bar = 2 mm and 50 μm (**w**).

Inspired by the potent immune responses induced by the 16a-HSA NE delivery system, we further investigated the tumor prevention efficiency. First, we treated mice according to the experimental protocol outlined in Fig. 5a to evaluate the efficacy of various immunization groups in preventing the initial subcutaneous challenge with EG7.OVA cells. As shown in Fig. 5b–i, among the groups immunized with the OVA antigen alone, the 16O group exhibited the most effective tumor suppression. In this group, only a few mice showed tumor growth by day 26 after tumor inoculation, whereas in the other control groups including PBS, FO (free OVA group), 0O, nearly all mice had tumors growing by this time, and most had succumbed to tumor overload. The median survival time for the PBS, FO, and 0O groups was 28 days, whereas the 16O group had a survival rate of approximately 56 %. In the immunization groups co-loaded with OVA and diABZI, 2 mice of FOA (free OVA and diABZI group) and 0OA group showed tumor growth respectively and succumbed successively between the 36th and 47th days after tumor inoculation. In contrast, the 16OA group showed no tumor growth within 94 days, suggesting that the 16OA held a potential advantage in the co-loaded group. Subsequently, we conducted multiple tumor cell challenges on the remaining surviving mice in each group to further assess their antitumor efficacy (Fig. 5a). According to survival data (Fig. 5j), the median survival times of the 0O, 16O, FOA, and 0OA groups were 28, 114, 238, and 240 days, respectively. In contrast, the 16OA group continued to protect 89 % of mice from tumor growth, demonstrating a significant advantage over the other groups. At the end of the observation period, we analyzed the spleen lymphocytes of the co-loading group mice (Fig. 5a). As seen in Fig. 5k–m, the 16OA group exhibited the highest levels of memory B cells and effector memory CD4^+^/CD8^+^ T cells. Additionally, the 16OA group had the lowest levels of immunosuppressive cells (Fig. 5n–p). Furthermore, after co-culturing spleen cells with antigens, we found that the 16OA group exhibited the strongest activation of CD4^+^/CD8^+^ T cells and NK cells (Fig. 5q–s).

Following the protocol outlined in Fig. 5t, we evaluated the efficacy of different immunization groups in preventing lung metastasis of B16-OVA tumors in mice. Among groups immunized with OVA alone, the 16O achieved most pronounced tumor suppression with only a few small lungs metastatic foci in some mice, while all mice in the other control groups (PBS, FO, 0O) exhibited extensive and numerous lung metastases (Fig. 5u–v), which was further confirmed by histological analysis (Fig. 5w). In the groups co-immunized with OVA and diABZI, 2 mice in FOA group and 1 mouse in 0OA group had lung metastasis, while the 16OA group had none, which was consistent with the prevention model above. This suggested that 16OA have a potential advantage in the co-loading group.

In summary, our findings fully highlighted the prospects of 16a-HSA NEs as a tumor prevention vaccine delivery vehicle. The 16O, containing only the antigen without additional adjuvants, still demonstrated strong tumor prevention. This effect primarily attributed not only to the excellent delivery efficiency of antigen but also to the inherent adjuvant properties of the vehicle. Additionally, its ER-targeted delivery also enhanced antigen cross-presentation and effectively initiates an antigen-specific cellular immune response. When co-delivered with the antigen and the extra adjuvant diABZI, accompanied by the enhanced effect of the precisely targeted delivery of diABZI, the 16OA formulation showed greater potential for tumor prevention.

### 2.6 Palmitic acid modification exhibited excellent in tumor therapy vaccine

**Fig. 6:**
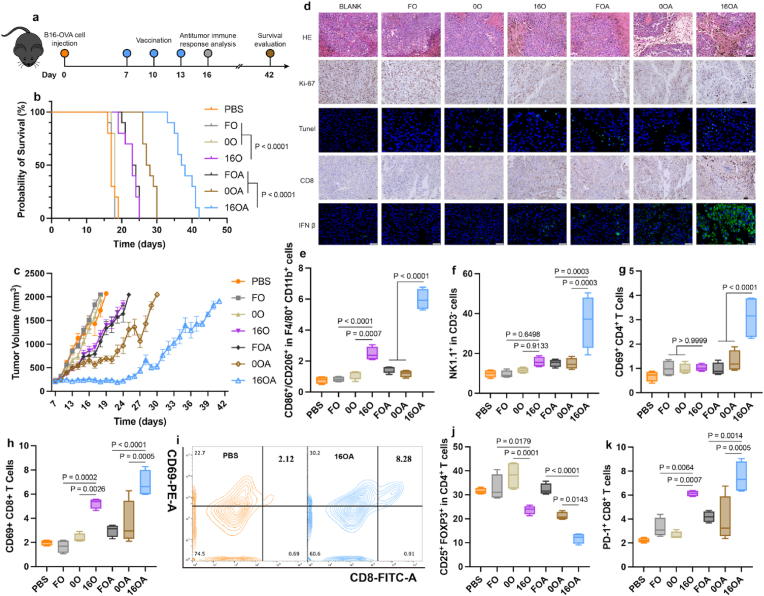
| 16a-HSA NEs Effectively Treat Tumors, Reshaping the Tumor Microenvironment. a, Mice were subcutaneously inoculated with B16-OVA tumor cells, followed by immunization with various vaccine formulations. Subsequent evaluations included systemic antitumor immune responses, tumor progression, and survival outcomes. **b**-**d**, Survival curves (**b**) (n = 9 per group), tumor growth curves (**c**) (n = 9 per group), and representative images of HE-stained, Ki-67 and CD8 immunohistochemically stained tumor sections, as well as Tunel and IFN β immunofluorescent staining (**d**) in mice treated with different vaccine formulations, scale bar = 50, 40, 20, 40, and 50 μm. **e**-**k**, Antitumor immune responses within the tumor microenvironment of mice from each immunization group (n = 4 per group): the M1/M2 macrophage ratio (**e**), the proportion of NK cells (**f**), activation levels of CD4^+^ T cells (**g**), activation levels of CD8^+^ T cells (**h**) accompanied by representative flow cytometry plots (**i**), the proportion of Treg cells (**j**), the proportion of CD8^+^ T cells expressing programmed cell death protein 1 (PD-1) (**k**).

Prophylactic vaccination is only applicable to tumors with specific antigens in populations with high incidence rates. Therefore, we further investigated the efficacy of 16a-HAS delivery vehicle in tumor therapy model. Following the protocol outlined in Fig. 6a, we evaluated the therapeutic effects of various immunization groups in mice bearing B16-OVA tumors. Despite inoculating a high tumor cell load of 1 × 10^6^ cells per mouse and initiating immunotherapy after tumors had grown to approximately 200 mm^3^, the survival and tumor growth curves still demonstrated the anti-tumor advantages of 16a-HSA NEs (Fig. 6b and c). The median survival times for the PBS, FO, 0O, 16O, FOA, 0OA, and 16OA groups were 17, 17, 18, 23, 23.5, 27.5, and 37.5 days, respectively. Compared to other groups immunized with OVA alone, the 16O group exhibited slower tumor growth and extended survival. Similarly, among the groups co-immunized with OVA and diABZI, the 16OA group also showed superior outcomes.

To make a more direct comparison of the effects of different vaccines, we further analyzed the tumor tissues. Histological analysis, including HE staining, Ki-67 immunohistochemistry, and Tunel immunofluorescence staining (Fig. 6d), revealed that in the OVA-only groups, the 16O group exhibited the best anti-tumor effects: necrotic tumor tissue accounted for about 30 % of the total, with approximately 7.05 % Tunel-positive and 12.88 % Ki-67-positive areas, indicating the highest levels of tumor necrosis and apoptosis and the lowest level of proliferation. In the groups co-immunized with OVA and diABZI, the 16OA group also showed the strongest anti-tumor effects, with necrotic areas comprising about 60 % of the tumor tissue, 20.25 % Tunel-positive, and 7.78 % Ki-67-positive areas. Additionally, immunohistochemical and fluorescence staining of tumor tissues revealed that the 16O and 16OA groups had the highest infiltration of CD8^+^ T cells and IFN-β in the single-loading group and the co-loading group, respectively (Fig. 6d).

We further assessed the immune modulation within the tumor microenvironment (Fig. 6e–k, Supplementary Figs. 21–25). The results indicated that in the co-delivery groups, the 16OA group exhibited the highest M1/M2 macrophage subtype ratio, the greatest NK cell infiltration, the strongest CD4^+^/CD8^+^ T cell activation, and the highest concentrations of proinflammatory cytokine such as IFN-β, IFN-γ, and TNF-α (Fig. 6e–i, and Supplementary Figs. 23–24). The 16OA group also showed the lowest levels of Treg cells (Fig. 6j). In the OVA-only groups, the 16O group similarly showed the highest M1/M2 macrophage ratio, the strongest CD8^+^ T cell activation, the highest cytokine concentrations, including IFN-β and IFN-γ, and the lowest Treg cell infiltration (Fig. 6e–h, j, and Supplementary Fig. 24). Meanwhile, treatment with 16a-HSA NEs (including the 16O and 16OA groups) resulted in elevated PD-1 expression on CD8^+^ T cells (Fig. 6k), paralleling their activation trend within the tumor microenvironment. This suggested that while the vaccine enhances immune responses, it may also activate immune checkpoint pathways, potentially increasing the risk of immune evasion and thereby attenuating the overall therapeutic efficacy. Therefore, in such a context, combining the vaccine with PD-1 inhibitors (e.g., anti–PD-1 antibodies) may represent an effective strategy to enhance antitumor efficacy. Correspondingly, the expression of PD-L1 on intratumoral cells was also upregulated following treatment with 16a- HSA NEs (Supplementary Fig. 25), further supporting the rationale for their combination with PD-1 blockade therapy.

**Fig. 7:**
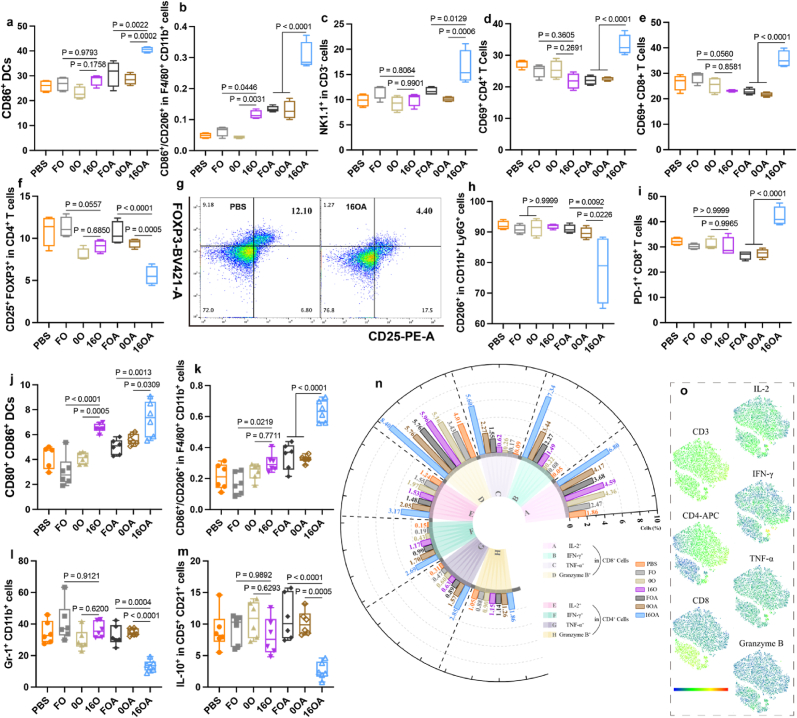
**16a-HSA NEs Induced Systemic Anti-Tumor Immune Response. a-i**, Antitumor immune responses within the lymph nodes of mice from each immunization group (n = 4 per group): DC maturation (**a**), the M1/M2 macrophage ratio (**b**), the proportion of NK cells (**c**), activation levels of CD4^+^ T cells (**d**), activation levels of CD8^+^ T cells (**e**), the proportion of Treg cells (**f**) accompanied by representative flow cytometry plots (**g**), N2-like neutrophils (**h**), and the proportion of CD8^+^ T cells expressing programmed cell death protein 1 (PD-1) (**i**). **j**-**o**, Antitumor immune responses within the spleens of mice from each immunization group (n = 6 per group): DC maturation (**j**), the M1/M2 macrophage ratio (**k**), the proportion of MDSC cells (**l**), the proportion of Breg cells (**m**), and the expression levels of IFN-γ, Granzyme B, TNF-α, and IL-2 in CD4^+^ or CD8^+^ T cells (**n**) accompanied by the representative flow cytometry color map from the 16OA group (**o**).

To elucidate the mechanism underlying the excellent anti-tumor effect of 16a-HSA NEs, we examined the systemic anti-tumor immune response in the mice. In the tumor-draining axillary lymph nodes, the 16OA group showed the strongest DC maturation, the highest M1/M2 macrophage subtype ratio, the greatest NK cell infiltration, the strongest CD4^+^/CD8^+^ T cell activation, and the lowest infiltration of Treg cells and N2-type neutrophils (Fig. 7a–h and Supplementary Fig. 26). Moreover, although 16OA treatment markedly enhanced the activation of CD8^+^ T cells in tumor-draining lymph nodes, it is noteworthy that PD-1 expression on their surface was simultaneously upregulated (Fig. 7i). This positive correlation between immune cell activation and PD-1 expression, consistent with observations in the tumor microenvironment, further reinforces the theoretical rationale for combining anti–PD-1 antibodies to optimize immunotherapeutic efficacy. Similarly, in the spleen lymphocytes, 16OA group exhibited the strongest DC maturation, the highest M1/M2 macrophage ratio, and the lowest levels of immunosuppressive MDSC cells (Fig. 7j–l and Supplementary Figs. 27–28). After co-culturing spleen lymphocytes with antigen stimulation and cytokine secretion blockers, the results showed that 16OA induced higher levels of IL-2, IFN-γ, TNF-α, Granzyme B in CD4^+^/CD8^+^ T cells (Fig. 7n and o, and Supplementary Fig. 29), higher memory B cell levels (Supplementary Fig. 30), and lower immunosuppressive Breg levels (Fig. 7m). The reduced levels of Breg partially support the advantages of diABZI for cell-specific delivery [31].

Then, cytokine measurements in the supernatant after co-culturing spleen lymphocytes with antigen showed a consistent trend with tumor cytokine levels (Supplementary Fig. 31). The 16OA group had the highest concentrations of IFN-β and TNF-α in the co-delivery groups, while the 16O group had the highest IFN-β concentration in the single-delivery groups. Body weight curves and histological analysis of major organs indicated that all formulations exhibited good biocompatibility *in vivo* (Supplementary Figs. 32–33). Overall, 16a-HSA NEs could combat tumors efficiently by inducing a potent systemic-specific immune response, and they also demonstrated high safety with no significant toxic side effects.

**Fig. 8:**
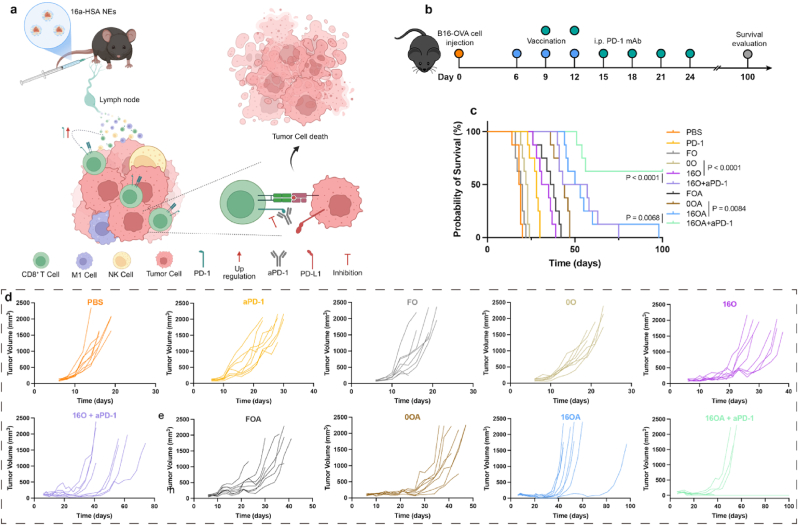
**The combination of 16a-HSA NEs and aPD-1 for tumor therapy. a**, Schematic illustration of the mechanism underlying the combination therapy. Created with BioRender.com. **b**, Schematic diagram of the experimental procedure: Mice were subcutaneously inoculated with B16-OVA tumor cells, followed by immunization with various vaccine formulations. In groups receiving 16a-HSA NEs, subsequent anti-PD-1 (aPD-1) therapy was administered. Tumor growth and survival were then evaluated. **c**, **d**, Survival curves (**c**) (n = 8 per group), tumor growth curves (**d**) (n = 8 per group).

Finally, the initial tumor volume at the time of administration was set at approximately 100 mm^3^. Notably, as 16a-HSA NEs have been observed to upregulate PD-1 expression on CD8^+^ T cells within tumors and tumor-draining lymph nodes, we investigated the therapeutic potential of combining 16a-HSA NEs with PD-1 blocker (Fig. 8a and b). As shown in Fig. 8c, d, 16a-HSA NEs exhibited a more pronounced antitumor effect compared to results obtained when treated at 200 mm^3^: the median survival times for the PBS, FO, 0O, 16O, FOA, 0OA, and 16OA groups were 18.5, 19.5, 22.5, 33, 37, 43.5, and 51.5 days, respectively. Furthermore, when anti-PD-1 antibody was co-administered with 16a-HSA NEs, the median survival time of the 16O group was extended to 50 days, while the 16OA group achieved complete tumor clearance and long-term survival in 62.5 % of tumor-bearing mice. These improvements were statistically significant when compared with 16a-HSA NEs monotherapy, demonstrating the considerable advantage of combining 16a-HSA NEs (particularly 16OA) with PD-1 blockade in cancer immunotherapy.

## 3 Conclusion

Subunit vaccines have recently garnered widespread attention due to their advantages in safety and controllability. In the design of subunit vaccines, appropriate vaccine delivery strategies can profoundly influence the *in vivo* and intracellular transport behavior of the vaccines, ultimately determining their efficacy. However, to date, there are few precedents for the clinical translation of nano-targeted delivery vectors in the context of subunit vaccines. Consequently, designing an efficient and biocompatible delivery vector for subunit vaccines remains a significant challenge, particularly when aligned with immunological principles. Given the substantial evidence underscoring the critical role of FAs in immune regulation, this study screened a series of FA-HSA nanocarriers and identified the promising 16a-HSA NEs as a potential delivery vehicle for subunit vaccines.

This study demonstrated that 16a-HSA NEs can efficiently drain to lymph nodes and are readily taken up by APCs, especially DCs and macrophages. Upon cellular entry, the 16a-HSA NEs exhibit a tendency to accumulate in the ER, an organelle critical for immune activation, thereby demonstrating their ability to facilitate precise, cascade delivery from organ to cell to organelle *in vivo*. Previous research has predominantly focused on organ and cellular levels, often overlooking the ER, a pivotal site for antigen cross-presentation. Subunit antigens generally exhibit weak immunogenicity and therefore require adjuvants to enhance their immune response. To meet clinical needs, there is an urgent demand for the development of adjuvant-based subunit vaccines, which necessitates the precise co-delivery of both antigen and adjuvant components. The ER serves as a critical site for antigen cross-presentation and the residence of STING. Our subsequent findings illustrate that the cascade delivery strategy enhanced the efficiency of antigen protein cross-presentation and STING activation on DCs, thereby maximizing the delivery and utilization of both antigen and adjuvant. This approach induces a robust, antigen-specific immune response characterized by increased antibody production, enhanced immune efficacy across various cell types (e.g., DCs, macrophages, T cells, B cells, and NK cells), upregulation of multiple anti-tumor cytokines, induction of systemic immune responses, and reshaping of the tumor microenvironment, ultimately exhibiting outstanding preventive and therapeutic efficacy in tumor models expressing the corresponding antigens. Moreover, we discovered that 16a-HSA NEs function not only as targeted delivery carriers but also as self-adjuvants. Transcriptomic analyses revealed that 16a-HSA NEs provided a sufficient transcriptional basis for enhancing antigen presentation and DCs maturation by upregulating key genes across multiple immune pathways. In summary, the use of endogenous components such as HSA and the non-essential fatty acid (16a) to construct a safe and efficient nano-delivery system offers a promising strategy for cascade targeting and maximum immune response mobilization.

Besides, our nano-delivery system exhibited significant potential for broader applications in disease prevention and treatment. It facilitated cascade delivery with multi-node amplification of therapeutic effects while utilizing safe, naturally derived materials that may reduce or even eliminate off-target effects and material-associated toxicities in clinical settings. We anticipate that similar tiered targeting delivery systems can be adapted for vaccines against various diseases by incorporating corresponding antigen proteins, such as those associated with malignancies caused by autologous cell mutations, bacterial infections, and viral infections. Collectively, our findings suggest that 16a-HSA NEs, as biomimetic nanocarriers for cascade targeted delivery, provide an efficient and safe strategy for the prevention and treatment of tumors expressing the model antigen protein OVA. This therapeutic strategy shows considerable potential in addressing the challenges associated with clinical translation and may offer broad applicability in the treatment of tumors and infectious diseases caused by various pathogens.

## 4 Materials and methods

### 4.1 Materials

Ovalbumin (OVA) from chicken was purchased from Sigma-Aldrich. diABZI was purchased from MedChemExpress. acetic acid-N – hydroxy-succinimide ester, caproic acid-N-hydroxy-succinimide ester, Octanoic acid-N- hydroxy-succinimide ester, Decanoic acid-N- hydroxy-succinimide ester, Myristic acid N-hydroxy-succinimide ester, palmitic acid-N-hydroxy-succinimide ester and stearic acid-N-hydroxy-succinimide ester were obtained from Yuanye Biotechnology, Lauric acid-N-hydroxy-succinimide ester and oleic acid-N-hydroxy-succinimide ester were obtained from Bidepharm. Human serum albumin (HSA) was purchased from Yuanda Shuyang. DiD was purchased from UElandy. CY5 NHS ESTER and LysoTracker Green DND-26 was purchased from MeilunBio®. TRITC [5(6)-Tetramethy rhodamine isothiocyanate] was purchased from Aladdin. Granulocyte–macrophage colony-stimulating factor (GM-CSF) was purchased from PeproTech. ER-Tracker Green was purchased from KeyGEN BioTECH. DAPI solution (ready-to-use) was purchased from Solarbio®. The following antibodies were used for Western blot analysis: IFN-β1 (D2J1D) rabbit mAb (catalogue number 97450S; clone name, D2J1D; 1:1000) was purchased from Cell Signaling Technology; β-Actin rabbit mAb (catalogue number 52901; clone name, unknown; 1:5000) was purchased from Signalway Antibody; HRP goat anti-rabbit IgG (H + L) antibody (catalogue number K1223; clone name, unknown; 1:10000) was purchased from APExBIO; Immobilon® Western chemiluminescent HRP substrate was purchased from Millipore. The following antibodies were used for determination of antibodies in serum: goat anti-mouse IgG H&L (HRP) (catalogue number ab6789; 1:50000), goat anti-mouse IgG1 (HRP) (catalogue number ab97240; 1:50000) and goat anti-mouse IgG2a heavy chain (HRP) (catalogue number ab97245; 1:50000) were purchased from Abcam. carboxyfluorescein succinimidyl aminoester (CFSE) was purchased from Merck. SIINFEKL peptide was purchased from GenScript. Mouse IL-1β uncoated ELISA kit (catalogue number 88-7013A-88), mouse mouse IL-4 uncoated ELISA kit (catalogue number 88-7044-88), mouse mouse IL-6 uncoated ELISA kit (catalogue number 88-7064-88), IFN-γ uncoated ELISA kit (catalogue number 88-7314-88), and mouse TNF-α uncoated ELISA kit (catalogue number 88-7324-88) were purchased from Invitrogen. Mouse IFN-β ELISA Kit was purchased from Ruixinbio. ELISpot Plus: Mouse IFN-γ (ALP) kit and ELISpot Plus: Mouse IL-4 (ALP) kit were purchased from Mabtech. Mouse lymphocyte isolation medium and serum-free medium (ELISPOT) were purchased from Dakewe. Flow intracellular fixation permeabilization buffer and Foxp3/transcription factor flow fixation permeabilization buffer were purchased from Thermo Fisher. Percoll was purchased from Cytiva. Antibodies used for flow cytometry are listed in Supplementary Table 4.

### 4.2 Cells and animals

DC2.4 cells provided from the Third Military Medical University were cultured with RPMI 1640 (Gibco) supplemented with 10 % FBS. RAW264.7 cells purchased from the American Type Culture Collection (ATCC) were cultured in high-glucose Dulbecco's modified Eagle's medium (DMEM, Gibco) supplemented with 10 % FBS. E.G7-OVA and B16-OVA cells both purchased from the American Type Culture Collection (ATCC) were cultured in RPMI 1640 (Gibco) supplemented with 10 % FBS and 0.4 mg/mL G418 sulfate. All the cell lines were cultured at 37 °C and 5 % CO2 in a humidified atmosphere. Female C57BL/6 mice (8 weeks old) were obtained from GemPharmatech. Mice were housed in a specific pathogen free state (20–24 °C with 40–70 % humidity and a 12 h day–night cycle) in the college's animal laboratory.

### 4.3 Synthesis and characterization

30 mg of lyophilized HSA powder (0.05 mmol) was completely dissolved in 10 mL of sodium bicarbonate solution (pH = 8.5). Each fatty acid-N-hydroxysuccinimide ester (FA-NHSE, 1 mmol, FA included 2a, 6a, 8a, 10a, 12a, 14a, 16a, 18a, and Olea) was fully dissolved in 1 mL of N, N-dimethylformamide (DMF). Under stirring at 37 °C, the FA-NHSE solution was added to the HSA solution and reacted for 24 h. The reaction mixture was then transferred to a dialysis bag (10 kDa) and dialyzed against deionized water for 60 h. After centrifugation to remove precipitates, the supernatant was freeze-dried to obtain a series of FA-HSA conjugates.

HSA and FA-HSA were dissolved in PBS (0.01 M, pH 7.2–7.4) buffer and subjected to fluorescence scanning using a microplate reader (Thermo Fisher 902-ULTS) at an excitation wavelength of 280 nm and emission wavelengths from 298 to 525 nm. Both HSA and FA-HSA were also dissolved in a series of citric acid buffers (pH 2.5–5.5) to determine their isoelectric points using a laser particle size analyzer (Malvern Zetasizer Nano ZS90). The degree of substitution of FA-HSA was quantified using the ninhydrin method. Specifically, a series of HSA and FA-HSA solutions at varying concentrations were added with ninhydrin solution, heated, and then cooled to room temperature. The resulting mixtures were diluted with a 60 % ethanol solution, and absorbance at 570 nm was measured using the microplate reader (Thermo Fisher 902-ULTS). The degree of substitution for FA-HSA was calculated using Supplementary formula 1, and its molecular weight was calculated using Supplementary formula 2. Additionally, Matrix-assisted laser desorption ionization tandem time-of-flight (MALDI-TOF) mass spectra of HSA and 16a-HSA were collected using a MALDI-TOF/TOF mass spectrometer (Shimadzu AXIMA Performance).

### 4.4 Formulation preparation and characterization

To prepare the formulation, 3.5 mg of soybean oil was dissolved in 0.2 mL of dichloromethane. Subsequently, 1 mL of HSA or FA-HSA solution (20 mg/mL) and 40 μL of antigen protein OVA solution (endotoxin-free, 10 mg/mL) were added. The mixture was subjected to probe sonication (200 W) for 5 min, and the organic solvent was removed via rotary evaporation. The solution was then adjusted to a final volume of 1 mL with sterile water, resulting in NEs containing OVA. Simultaneously, 1.5 μL DMSO solution containing diABZI (16 mg/mL) was added to dichloromethane to obtain 0OA or 16OA; Additionally, 12.5 μg DiD was added to dichloromethane to obtain NEs containing DiD; Similarly, OVA was replaced by OVA-Cy5 or OVA-TRITC to obtain NEs containing CY5 or TRITC.

The particle size, polydispersity index (PDI), and zeta potential of the NEs were measured using a laser particle size analyzer (Malvern Zetasizer Nano ZS90). Transmission electron microscopy images of 0O, 16O, 0OA, and 16OA were also captured. Unencapsulated free drugs or NEs were separated using ultrafiltration tubes or dextran gel columns. The content of the free drug ($W_{Free}$), the drug content encapsulated in the nano-emulsion ($W_{NEs}$), and the total drug content in the formulation ($W_{Total}$) were subsequently quantified using fluorescence quantitative analysis (OVA-Cy5) or high-performance liquid chromatography (diABZI). The encapsulation efficiency (EE) of OVA or diABZI was then calculated according to the Supplementary formula 3. Furthermore, the stability of the formulations was evaluated by assessing the particle size of the NEs at different temperatures (4 °C and 37 °C) over time. Additionally, the formulations were co-cultured with 10 % serum at 37 °C with gentle shaking (50 rpm) to study their stability, measuring both particle size and transparency (T). T was calculated using the Supplementary formula 4.

### 4.5 Culture of BMDCs

Bone marrow was isolated from the femurs and tibias of C57BL/6 mice and washed with cold RPMI 1640 medium. The bone marrow cells were filtered through a cell strainer and resuspended in RPMI 1640 medium containing mouse recombinant granulocyte-macrophage colony-stimulating factor (GM-CSF, 20 ng/mL), 2-mercaptoethanol (50 μM), fetal bovine serum (FBS, heat-inactivated, 10 %), and penicillin-streptomycin solution (1 %). Cells were seeded at a density of 2 × 10^7^ cells per dish, recorded as day 0. On day 3, an equal volume of fresh medium was added. On days 5–6, non-adherent cells were collected by centrifugation, yielding primary bone marrow-derived cells.

### 4.6 Uptake of formulations by different APCs and inhibition studies

In the uptake experiments with BMDCs, the primary bone marrow-derived cells were seeded at a density of 1 × 10^6^ cells per well in a cell culture plate and cultured for 2–8 h. Subsequently, 25 μL of HSA and FA-HSA NEs containing DiD were added respectively, followed by 1 h of incubation. The BMDCs were labeled with anti-mouse CD11c-BB700 and analyzed using a flow cytometer (BD FACSCelesta).

For uptake experiments with DC2.4 and RAW264.7 cells, cells were seeded at a density of 1 × 10^6^ cells per well and cultured overnight. Following this, 25 μL of 0O and 16O containing DiD or OVA-Cy5 were added respectively, and cells were incubated for 1 h before measurement. For the uptake inhibition experiments in DC2.4 cells, the procedure was similar, but cells were pre-treated with various inhibitors or under specific conditions (details provided in the Supplementary Table 5) for 1 h before adding 0O or 16O containing DiD.

### 4.7 Co-localization of formulations with ER/lysosome in DC2.4 cells

DC2.4 cells were seeded at a density of 3 × 10^5^ cells per well in a confocal dish and cultured overnight. Subsequently, 25 μL of 0O or 16O containing DiD were added and incubated for 1 h. The cells were then treated with 1 mL of ER-Tracker Green/LysoTracker Green DND-26 working solution (400 nM/50 nM) and incubated for an additional 30 min. After fixation, nuclei were stained with DAPI, and the co-localization of 0O and 16O with the ER was observed using a super-resolution laser confocal microscope (Zeiss LSM 800).

### 4.8 Flow cytometry assay

The flow cytometry experimental protocol began with resuspending the collected cells in the Fixable Viability Dye eFluor™ 506 working solution. The cells were then incubated at 4 °C in the dark for 30 min. Following this, the cells were washed and preincubated with Mouse BD Fc Block™ at 4 °C for 20 min. Specific cell surface antibodies were subsequently added directly to the preincubated cells, in the presence of Mouse BD Fc Block™, and incubated at 4 °C for 40 min. After washing, the stained cells are filtered and analyzed using a flow cytometer (BD FACSCelesta). For intracellular staining, the cells are treated with an intracellular fixation buffer and resuspended in the intracellular flow antibody staining solution. After staining and washing, the cells are filtered prior to analysis. For nuclear staining, the cells are treated with Foxp3 fixation/permeabilization working solution and resuspended in the nuclear flow antibody staining solution. Following staining, washing, and filtering, the cells are analyzed. Data are processed using FlowJo v.10 software (TreeStar).

### 4.9 Activation of BMDCs and T cells by the formulations

For the antigen cross-presentation and maturation assay, primary bone marrow-derived cells were seeded at a density of 1 × 10^6^ cells per well and cultured for 2–8 h. Subsequently, 25 μL of the formulation solution was added and incubated for 20 h. In some cases, LPSO, a free mixture of LPS and OVA (with an LPS dose of 5 μg per well), was used as the positive control group. Staining and determination were performed according to flow cytometry protocol. We used the following markers to identify cell populations: antigen cross-presentation (CD11c^+^ H2Kb-SIINFEKL^+^) and maturation (CD11c^+^ CD40^+^ CD80^+^).

For the *in vitro* T cell activation, the BMDCs were first treated with indicated formulations for 24 h, then followed by co-culture with splenic lymphocytes extracted from naïve mice for 48h.T cell activation was analyzed by flow cytometry using activation markers (e.g., CD69), and the proliferation of T cells is characterized by the increase in the proportion of CD3^+^ T cells, while Granzyme B secretion in the co-culture supernatants was measured via ELISA to assess T cell cytotoxicity and functional responses.

For Western blotting (WB) to assess STING activation, the sample preparation followed the same steps as above, but after culturing, cells were resuspended in RIPA lysis buffer containing PMSF. The protein concentration of each lysate was measured using a BCA kit and adjusted to be uniform. SDS-PAGE sample buffer and lysate were mixed and heated for denaturation. Subsequently, a 4.5–10 % Omni-Easy PAGE gel was prepared, and denatured samples were loaded for protein separation via electrophoresis. Proteins were then transferred to a PVDF membrane at 100 V. The membrane was washed with TBST and blocked with TBST containing 5 % nonfat milk. After washing, the membrane was cut reasonably according to marker molecular weight bands and incubated overnight at 4 °C with corresponding primary antibodies. Following washing, secondary antibodies were added and incubated at 37 °C for 1 h. The membrane was washed again, and ECL detection solution was applied. β-Actin served as a control, and images were captured and analyzed using the gel imaging system and Image Lab 6.1 (Bio-Rad).

### 4.10 Lymph node targeting and distribution

C57BL/6 mice received a subcutaneous injection of 25 μL of 0O and 16O containing Cy5-OVA or DiD, as well as free Cy5-OVA or DiD. Mice were euthanized at 2, 6, 12, and 24 h post-injection. The ipsilateral popliteal lymph nodes were harvested, and their fluorescent images were captured using a small animal optical imaging system (PerkinElmer IVIS Lumina III). Quantitative analysis of the fluorescence was performed using Living Image software. In a parallel experiment, following the injection of 0O and 16O containing Cy5-OVA or TRITC-OVA and DiD, as well as free Cy5-OVA or TRITC-OVA and DiD (FO), mice were euthanized at 6 h post-injection. The ipsilateral popliteal lymph nodes were collected for frozen sectioning. After DAPI staining for nuclei, imaging was conducted using a super-resolution laser scanning confocal microscope (Zeiss LSM 800) to examine the distribution of each formulation within the popliteal lymph nodes. Additionally, the ipsilateral inguinal lymph nodes were also harvested, and fluorescence intensity images were collected using the small animal optical imaging system (PerkinElmer IVIS Lumina III). This assessment aimed to evaluate the targeting efficiency of each formulation in draining to more distant lymph nodes.

For further analysis, C57BL/6 mice were injected with 25 μL of 0O and 16O containing Cy5-OVA, as well as free Cy5-OVA, and then euthanized at 6 h post-injection. The popliteal lymph nodes were processed to obtain a cell suspension, which was stained and determined according to flow cytometry protocol. The following markers were used to identify cell populations: dendritic cells (DCs, CD11c^+^), cDC1 (CD8a^+^ DCs), cDC2 (CD11b^+^ DCs), macrophages (F4/80^+^), B cells (CD19^+^), neutrophils (Ly6G^+^), and T cells (CD3^+^), to investigate the uptake of various formulations by different cell types.

### 4.11 STING pathway activation *in vivo*

C57BL/6 mice received a subcutaneous injection of 25 μL of PBS, FO, 0O, 16O, FOA, 0OA, or 16OA solutions. After 60 h, mice were euthanized, and the inguinal lymph nodes on the injection side were harvested and were homogenized in RIPA lysis buffer using a cryogenic grinder. The homogenates were centrifuged at 12,000 rpm for 10 min at 4 °C, and the supernatant was collected as the total protein extract. A portion of the protein was denatured in loading buffer and subjected to Western blot analysis to detect p-TBK1 expression. The remaining protein was used for ELISA to quantify IFN-α and IFN-β levels.

### 4.12 DC activation in lymph nodes

C57BL/6 mice received a subcutaneous injection of 25 μL of PBS, FO, 0O, 16O, FOA, 0OA, or 16OA solutions. After 60 h, mice were euthanized, and the inguinal lymph nodes on the injection side were harvested to prepare cell suspensions for flow cytometry analysis. The following markers were employed to assess DC activation *in vivo* induced by different formulations: antigen cross-presentation (CD11c^+^ H2Kb-SIINFEKL^+^), and maturation (CD11c^+^ CD40^+^ CD86^+^).

### 4.13 Detection of specific antibody levels in serum

Mice serum was obtained at the scheduled time, and the specific antibody levels in serum were detected by enzyme-linked immune sorbent assay (ELISA). Briefly, OVA was dissolved in coating buffer (10 μg/mL) and added (100 μL per well) to the ELISA plate (Costar 9018) overnight at 4 °C. The next day, the plate was washed and blocked with blocking solution at 37 °C for 1 h. Serum samples were diluted in a gradient, usually across 8–12 dilutions, followed by washing. Diluted samples were added to the plate and incubated overnight at 4 °C. After several washes, HRP-conjugated secondary antibodies were added according to the manufacturer's instructions and incubated at 37 °C for 1 h. Following additional washes, TMB substrate was added. When the blue color was obvious, the termination solution was added and read at 450 nm.

### 4.14 Activation of T/B cells in mouse lymph nodes

In accordance with the experimental schedule, inguinal lymph nodes were harvested from mice and prepared as cell suspensions. Cells were seeded at a density of 1.5 × 10^6^ cells per well in 96-well plates, and OVA and SIINFEKL peptide were added as stimuli. After 48 h of culture, flow cytometry was performed using the following markers: T cell activation (CD3^+^ CD4^+^ CD69^+^,CD3^+^ CD8^+^ CD69^+^), memory B cells (CD19^+^ CD27^+^), and germinal center B cells (CD19^+^ B220^+^ GL7^+^) to investigate T/B cell activation in the lymph nodes.

### 4.15 Assessment of CTL killing efficiency

The experiment requires two days to complete. On Day 1, euthanize a suitable number of healthy C57BL/6 mice according to experimental needs. Harvest the spleens and prepare a cell suspension by grinding the spleens. Use red blood cell lysis buffer to lyse the cells, then resuspend them in a 1640 culture medium containing serum. Distribute the cell suspension evenly into two culture dishes. Add the SIINFEKL antigen peptide to “the target dish” (2 μg/ml) and an equal volume of PBS or a negative control peptide to “the reference dish”, culturing for 1 h. Wash the cells, then add a high concentration of CFSE dye to the target cells (5 μM), labeling them as CFSE^hi^, and a low concentration of CFSE dye to the reference cells (0.5 μM), labeling them as CFSE^low^, and incubate for 10 min. Add FBS to stop the staining and wash the cells again. Count the cells in both tubes, adjust their concentrations to match, and mix equal volumes. Inject the mixed cells via the tail vein into the immunized mice. On Day 2, euthanize the mice from each group, isolate the spleens, prepare a cell suspension, lyse red blood cells, and wash before analyzing with a flow cytometer (BD FACSCelesta). The specific lysis rate was calculated by the Supplementary formula 5.

### 4.16 Isolation of spleen lymphocytes

Mice were euthanized according to the experimental protocol, and spleens were excised and ground into the cell suspension using lymphocyte separation medium under light protection. The suspension was carefully transferred to a centrifuge tube, and serum-free medium was added along the wall of the tube to maintain the interface between the two liquids clear. After centrifugation, the lymphocyte layer was extracted, lysed, and washed before resuspending in 1 mL of ELISPOT-specific serum-free medium, counting the cells using an automatic cell counter (CountStar) to achieve a uniform concentration.

### 4.17 ELISPOT detection of IFN-γ and IL-4 secretion from spleen lymphocytes

Pre-coated ELISPOT plates for IFN-γ and IL-4 were washed with PBS, then incubated with specialized culture medium to activate the PVDF membranes at the bottom of the wells. The isolated splenic lymphocytes were inoculated into the ELISPOT plates (1 × 10^6^ or 2 × 10^6^ cells per well) and SIINFEKL peptide was added as stimulator, while the control wells were reasonably set up. After 21 h of culture, wells were washed and incubated with detection antibodies at room temperature for 2 h. Following additional washes, alkaline phosphatase-conjugated streptavidin was added and incubated for 1 h. After another series of washes, BCIP/NBT substrate was added, and the reaction was halted once visible spots appeared, allowing for quantification with an ELISPOT reader (Mabtech IRIS).

### 4.18 Intracellular cytokine analysis of splenic lymphocytes

Isolated spleen lymphocytes were seeded at a density of 1 × 10^7^ cells per well and stimulated with OVA and SIINFEKL peptide. A positive control was included, and after 1 h of culture, the protein transport inhibitor monensin was added to prevent cytokine secretion from the ER to the Golgi apparatus, allowing cytokines to accumulate within the cells. Following a further 5 h of culture, cells were washed, stained, and analyzed by flow cytometry using markers for T cells (CD3^+^ CD4^+^,CD3^+^ CD8^+^) and intracellular cytokines (IL-2^+^, IL-4^+^, IFN-γ^+^和TNF-α^+^).

### 4.19 Measurement of cytokines in culture supernatants of spleen lymphocytes

Spleen lymphocytes were seeded at a density of 1 × 10^7^ cells per well and co-cultured with OVA and SIINFEKL peptide for 72 h. Culture supernatants were collected and centrifuged. Supernatants were transferred to new tubes for cytokine measurement using uncoated mouse ELISA cytokine kits to detect levels of IL-1β, IL-4, IL-6, IFN-β, IFN-γ, and TNF-α.

### 4.20 Evaluation of therapeutic efficacy against tumors

Preventive Model with E.G7-OVA and B16-OVA: C57BL/6 mice were randomly grouped and immunized according to the protocol shown in Fig. 5a (OVA dosage: 10 μg/mouse; diABZI dosage: 0.6 μg/mouse) on days −21, −14, and −7. On day 0, each mouse received 1 × 10^6^ E G7-OVA cells subcutaneously in the right axilla. Mice were observed daily, and starting from day 6, tumor were measured using calipers to record the longest (l) and shortest (w) diameters. Tumor volume (V, mm^3^) was calculated using the Supplementary formula 6, and growth curves were plotted. Natural death dates were recorded for survival analysis. Based on ethical considerations, the following criteria were defined for euthanasia: (1) tumor length (l) exceeds 20 mm; (2) tumor volume exceeds 2000 mm^3^. On day 94, mice previously immunized with OVA and diABZI formulations (FOA, 0OA, 16OA) were injected subcutaneously with 1.5 × 10^6^ E G7-OVA cells, and monitored until day 155. Subsequently, mice received tail vein injections of 4 × 10^5^ B16-OVA cells and were observed until day 199, followed by a right axillary injection of 1 × 10^6^ B16-OVA cells and monitored until day 270. Remaining mice from the FOA, 0OA, and 16OA groups were euthanized, and spleens were harvested to prepare lymphocyte suspensions. One portion was stained for flow cytometry to identify populations including memory B cells (CD19^+^ CD27^+^), effector memory T cells (CD3^+^ CD4^+^ CD44^+^ CD62L^−^,CD3^+^ CD8^+^ CD44^+^ CD62L^−^), and immunosuppressive cells (CD3^+^ CD4^+^ CD25^+^ FOXP3^+^,CD3^+^ PD-1^+^,PD-L1^+^). The remaining portion was cultured with antigen for 72 h and stained for analysis of T cell activation (CD3^+^ CD4^+^ CD69^+^,CD3^+^ CD8^+^ CD69^+^) and NK cell proliferation (CD3^−^ NK1.1^+^).

B16-OVA Lung Metastasis Prevention Model: C57BL/6 mice were randomly grouped and immunized according to the scheme in Fig. 5t (OVA dosage: 10 μg/mouse; diABZI dosage: 0.6 μg/mouse) on days −14 and −7. On day 0, mice were injected via the tail vein with 5 × 10^5^ B16-OVA cells. On day 39, following natural deaths in the PBS group, all groups were euthanized to observe lung tumor metastasis. Images were taken, and the number of tumor metastases in the lungs was recorded. Representative lung tissues were sectioned and stained with HE.

B16-OVA Subcutaneous Large Tumor Treatment Model: C57BL/6 mice were randomly grouped and on day 0 received 1 × 10^6^ B16-OVA cells subcutaneously. When tumors reached approximately 200 mm^3^, mice were vaccinated according to the protocol in Fig. 6a (OVA dosage: 10 μg/mouse; diABZI dosage: 0.6 μg/mouse) on days 7, 10, and 13. The longest diameter (l) and shortest diameter (w) of the tumor were measured by vernier caliper, and the tumor volume (V, mm^3^) was calculated by the Supplementary formula 6, and the body weight of the mice was weighed. On day 16, some mice in each group were euthanized to collect spleen, tumor, axillary lymph nodes, heart, liver, lung, and kidney tissues. Preparation of tumor cell suspensions was based on previously reported methods [57,58], in brief: tumors were cut into small pieces, ground in HBSS buffer containing DNAse I (100 μg/ml, Invitrogen) to obtain cell suspensions, cells were counted, each sample is divided into an equal number of cells. After red blood cells were lysed, the cells were washed, stained and determined by flow cytometry. The remaining cells were separated by Percoll density gradient centrifugation to isolate tumor-infiltrating lymphocytes, then pocessed by the flow cytometry protocol. We used the following markers to identify cell populations: lymphocytes (CD45^+^), macrophages M1 and M2 (F4/80^+^ CD11B^+^ CD206^-^ CD86^+^,F4/80^+^ CD11B^+^ CD206^+^), NK cells (CD3^−^ NK1.1^+^), activated T cells (CD3^+^ CD4^+^ CD69^+^,CD3^+^ CD8^+^ CD69^+^), Tregs (CD3^+^ CD4^+^ CD25^+^ FOXP3^+^), and cells expressing immune checkpoint molecules (PD-L1^+^, CD3^+^ CD8^+^ CD69^+^). Lymph node cells were also stained for DCs maturation (CD11c^+^ CD86^+^), macrophages M1 and M2 (F4/80^+^ CD11B^+^ CD206^-^ CD86^+^,F4/80^+^ CD11B^+^ CD206^+^), NK cells (CD3^−^ NK1.1^+^), T cell activation (CD3^+^ CD4^+^ CD69^+^,CD3^+^ CD8^+^ CD69^+^), Tregs (CD3^+^ CD4^+^ CD25^+^ FOXP3^+^), N2 type neutrophils (CD11b^+^ Ly6G^+^ CD206^+^), and cells expressing immune checkpoint molecules (CD3^+^ CD8^+^ CD69^+^). Spleen lymphocyte suspensions were divided into three parts: one for immediate flow cytometry including markers: DCs maturation (CD11c^+^ CD80^+^ CD86^+^), macrophages M1 and M2 (F4/80^+^ CD11B^+^ CD206^-^ CD86^+^,F4/80^+^ CD11B^+^ CD206^+^), MDSC (CD11b^+^ Gr-1^+^); the second for intracellular cytokine assays including markers: Breg (CD19^+^ CD1d^+^ CD5^+^ CD21^+^ IL-10^+^), T cells (CD3^+^ CD4^+^,CD3^+^ CD8^+^), intracellular cytokines (IL-2^+^, IFN-γ^+^, TNF-α^+^, Granzyme B^+^); and the third for antigen culture followed by flow cytometry to assess memory B cells (CD19^+^ CD27^+^), meanwhile the cell supernatant was collected for cytokine assays.

Tumor samples also were taken for measurement of cytokines. The specific method was as follows: tumor tissue was mixed with RIPA lysate (containing proteinase inhibitors) under the ablation using a tissue ablator (DocSense SC-G1). The supernatant was collected by centrifugation, and the protein concentration of each sample was determined using a BCA reagent kit. The concentrations of cytokines IFN-β, IFN-γ, and TNF-α were then determined using the ELISA kit. Part of the tumor tissue was also used for histological staining, immunohistochemical staining, and immunofluorescence staining, while the heart, liver, spleen, lung, and kidney were also used for histological staining. These experiments were assisted by Chengdu Lilai. The remaining mice were observed, and tumor volume growth curves and weight change curves were drawn, while the dates of natural death were recorded to draw survival curves.

B16-OVA Subcutaneous Medium Tumor Treatment Model: C57BL/6 mice were randomly grouped and on day 0 received 6 × 10^5^ B16-OVA cells subcutaneously. When tumors reached approximately 100 mm^3^, mice were vaccinated according to the protocol in Fig. 8a (OVA dosage: 10 μg/mouse; diABZI dosage: 0.6 μg/mouse) on days 6, 9, and 12. Besides, starting on day 9, mice in the PBS, 16O, and 16OA groups received intraperitoneal injections of anti-PD-1 monoclonal antibody (mAb) at a dose of 100 μg per mouse (0.1 mL) every three days, administered on days 9, 12, 15, 18, 21, and 24. From day 6, tumor volumes were measured regularly, mortality was monitored, and tumor growth and survival curves were generated accordingly.

### 4.21 Transcriptome sequencing

BMDCs were treated with various formulations for 20 h, followed by resuspension in TRIzol lysis reagent for subsequent RNA extraction. Briefly, the process involves isolating total RNA, enriching mRNA with Oligo dT, fragmenting mRNA, reverse transcribing cDNA, connecting adapter molecules, selecting fragments, and enriching the library, followed by sequencing on the NovaSeq X Plus (Illumina). The data were analyzed on www.majorbio.com.

### 4.22 Statistical analysis

All statistical analysis was performed by GraphPad Prism software (v.10.1.1.270). One-way ANOVA followed with Tukey's multiple-comparisons test was performed for data analysis. For mouse survival curves, data were analyzed via Kaplan-Meier analysis and log-rank (Mantel–Cox) test. Significance was considered if the P value was less than 0.05. The data are presented as the mean ± SD.

## CRediT authorship contribution statement

**Yuan Xue:** Writing – review & editing, Writing – original draft, Visualization, Validation, Supervision, Software, Project administration, Methodology, Investigation, Formal analysis, Data curation, Conceptualization. **Shuting Bai:** Writing – review & editing, Visualization, Validation, Methodology, Investigation. **Yating Wang:** Investigation. **Jiaxing Feng:** Investigation. **Kun Xiong:** Investigation. **Xue Tang:** Investigation. **Chunting He:** Investigation. **Yanhua Xu:** Investigation. **Hongling Yu:** Investigation. **Tianyi Luo:** Investigation. **Qing Lin:** Writing – review & editing, Resources. **Xun Sun:** Resources. **Ling Zhang:** Writing – review & editing, Resources. **Zhirong Zhang:** Resources. **Tao Gong:** Writing – review & editing, Supervision, Resources, Project administration, Funding acquisition, Conceptualization.

## Ethics approval and consent to participate

All animal experiments were performed under the guidelines evaluated and approved by the medical ethics committee of Sichuan University (Approved number: K2023037).

## Declaration of competing interest

The authors declare the following personal relationships which may be considered as potential competing interests: Yuan Xue is currently employed by Chengdu Origen Biotechnology Co. Ltd.
