# Supplementary material for: Cascade-targeted delivery platform enhances antigen cross-presentation and STING activation for durable cellular immunity
Source: Bioact Mater. 2025 Sep 3;54:584–601. doi: 10.1016/j.bioactmat.2025.08.033 (PMC12445615; doi:10.1016/j.bioactmat.2025.08.033)
Supplement: Multimedia component 1 [file mmc1.pdf]

Supplementary Materials for

Cascade-targeted delivery platform enhances antigen cross-presentation and STING activation for durable cellular immunity

This PDF file includes:

- Supplementary formula 1 to 5
- Supplementary Fig. 1 to 33
- Supplementary Table 1 to 5

Supplementary formulas

Supplementary formula 1 Formula for calculating amino substitution degree of lysine residues in FA-HSA.

$$DS_{FA-HSA} = \frac{C_{K(HSA)} - C_{K(FA-HSA)}}{C_{K(HSA)} + \alpha \times C_{K(FA-HSA)}}$$

DS_{FA-HSA} represents the degree of amino substitution on the lysine residues in each FA-HSA. $C_{K(HSA)}$ and $C_{K(FA-HSA)}$ represent the concentration of intact lysine residues of B mg HSA and FA-HSA dissolved in C mL deionized water, respectively. $\alpha = N \times (M_{FA} - 18)/M_{HSA}$, and N= 59 (The number of lysine residues in an HSA molecule), M_{FA} and M_{HSA} represent the molecular weights of FA and HSA, respectively.

Supplementary formula 2. Formula for calculating the molecular weight of each FA-HSA.

$$M_{FA-HSA} = M_{HSA} + (M_{FA} - 18) \times DS_{FA-HSA} \times N$$

M_{FA-HSA}, M_{HSA} and M_{FA} represent the molecular weight of FA-HSA, HSA and FA respectively. DS_{FA-HSA} represents the degree of amino substitution on the lysine residues in each FA-HSA. N= 59 (The number of lysine residues in an HSA molecule).

Supplementary formula 3. Encapsulation efficiency calculation formula.

$$EE\ (\%) = \frac{W_{NEs}}{W_{Total}} \times\ 100\% = \frac{1 - W_{Free}}{W_{Total}} \times 100\%$$

W_{NEs}, W_{Free} and W_{Total} represent the content of the free drug, the drug content encapsulated in the nano-emulsion, and the total drug content in the formulation.

Supplementary formula 4. Transparency calculation formula for each solution.

$$T\% = 10^{-A}$$

T is transparency, and A represents absorbance at 750 nm.

Supplementary formula 5. The specific lysis rate calculation formula.

$$specific\ lysis\ (\%) = (1 - \frac{ratio_E}{ratio_B}) \times 100\%$$

$ratio = the\ cell\ counts\ of\ CFSE^{hi}/the\ cell\ counts\ of\ CFSE^{low}$, E is experimental group, B is blank group。

Supplementary formula 6. Tumor volume calculation formula.

$$V = \frac{l \times w^2}{2}$$

V, l and w represent the tumor volume, the longest diameter and shortest diameter of

the tumor, respectively.

Supplementary figures

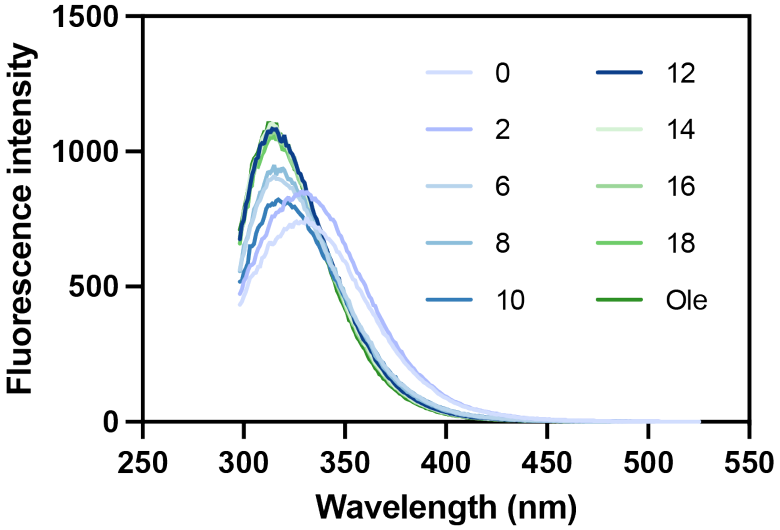

Supplementary Fig. 1 The fluorescence emission spectra of a series of HSA modified with fatty acids. Ex = 280 nm, Em = 298-525 nm.

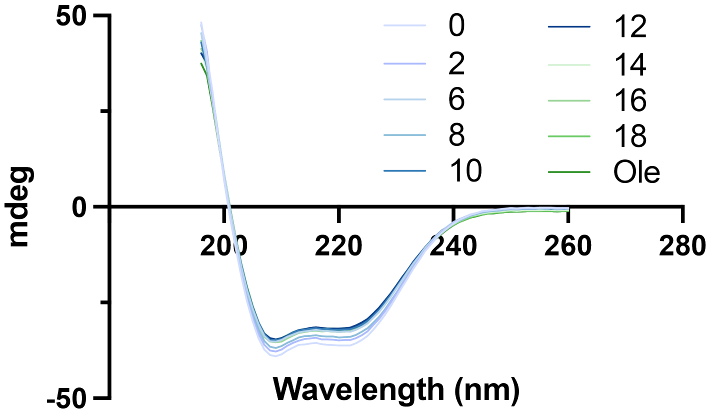

Supplementary Fig. 2 The Circular dichroism chromatography of a series of HSA modified with fatty acids.

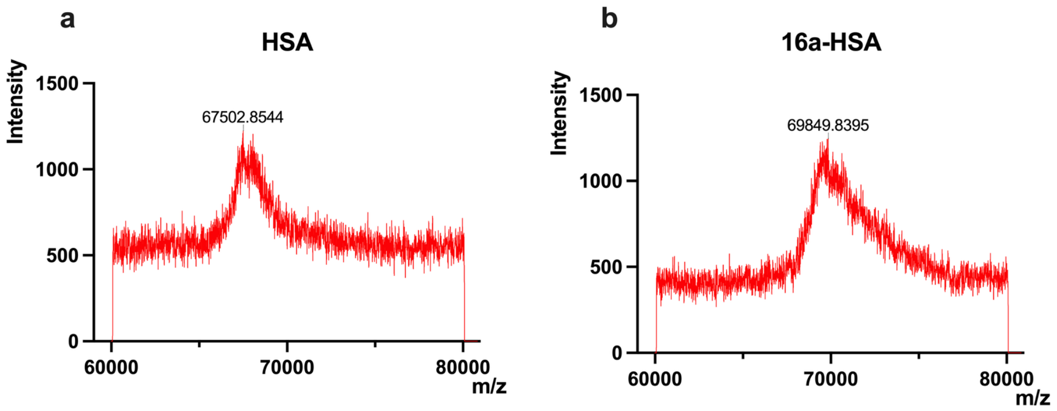

Supplementary Fig. 3 The matrix assisted laser desorption ionization time of flight mass spectrometry (MALDI-TOF) of HSA (**a**) and 16a-HSA (**b**).

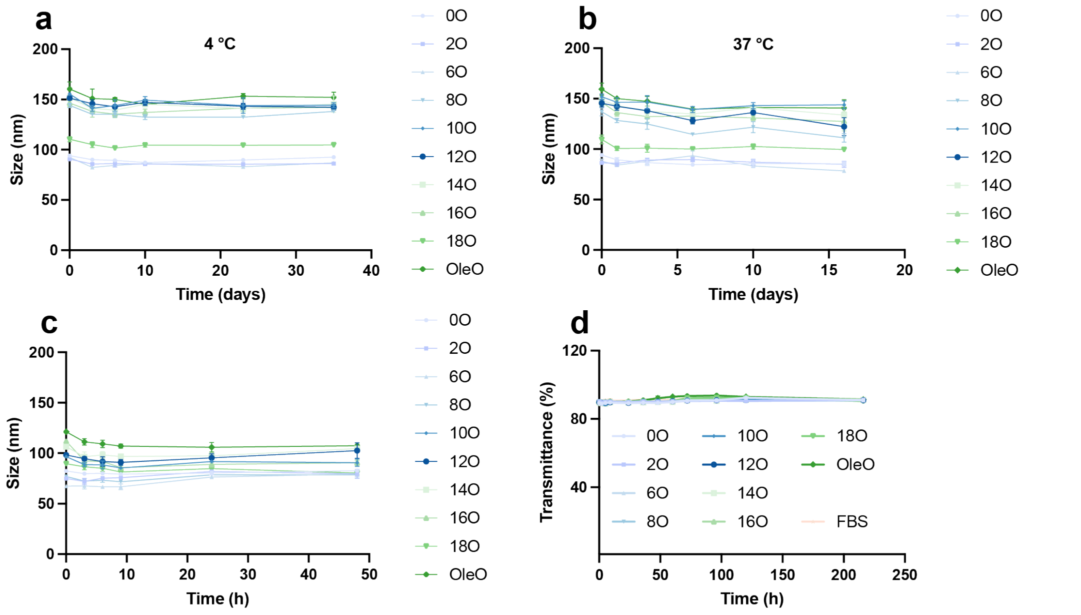

Supplementary Fig. 4 Characterization for the stability of preparation. **a**, The particle size curve of preparation stored under 4 °C (n = 3 per group). **b**, The particle size curve of preparation stored under 37 °C (n = 3 per group). **c**, The particle size curve of preparation co-incubated with 10% FBS under 37 °C (n = 3 per group). **d**, The transmittance curve of preparation co-incubated with 50% FBS under 37 °C (n = 5 per group).

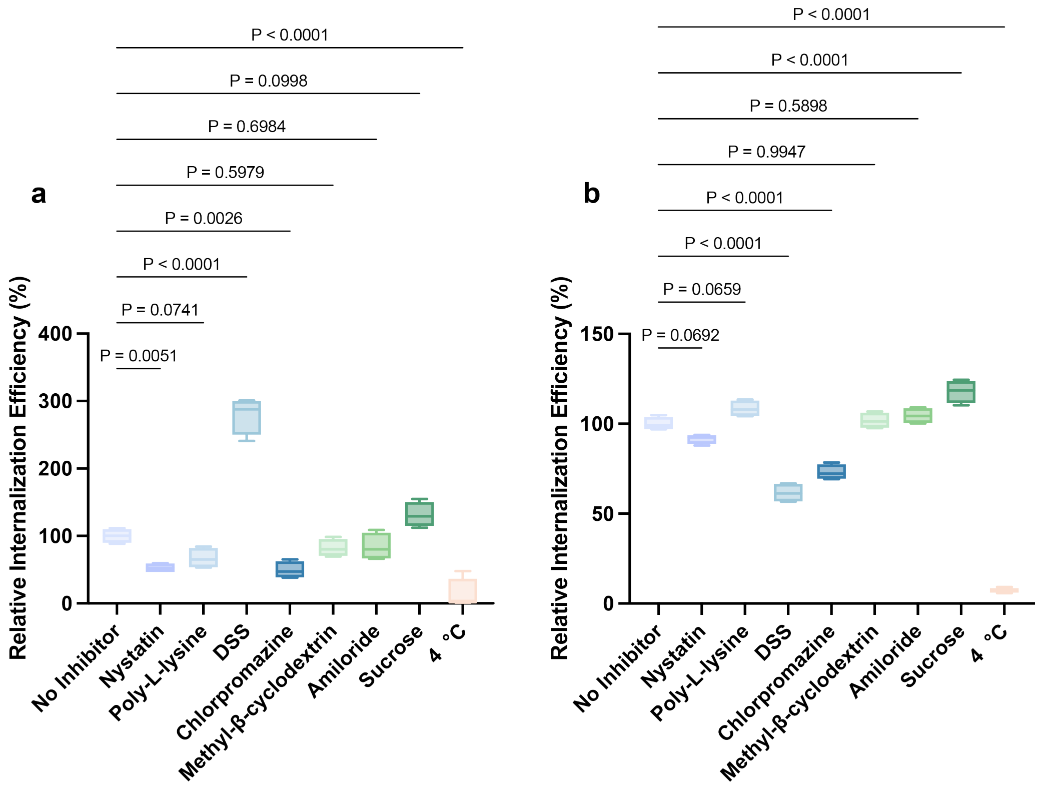

Supplementary Fig. 5 The uptake inhibition assay of (**a**) 0O and (**b**) 16O on DC2.4 (n = 4 per group).

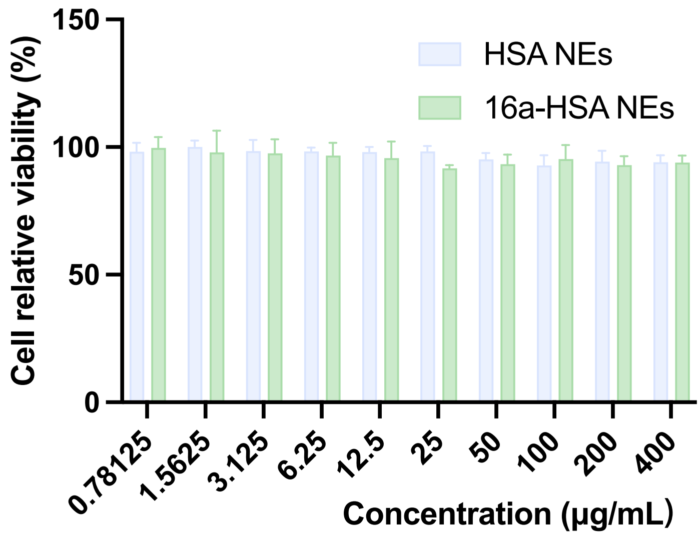

Supplementary Fig. 6 DC2.4 cells were treated with a series of concentration gradients of blank nano-emulsions (NEs), and cell viability was assessed after 24 hours of co-incubation using the CCK-8 assay kit (n = 5 per group).

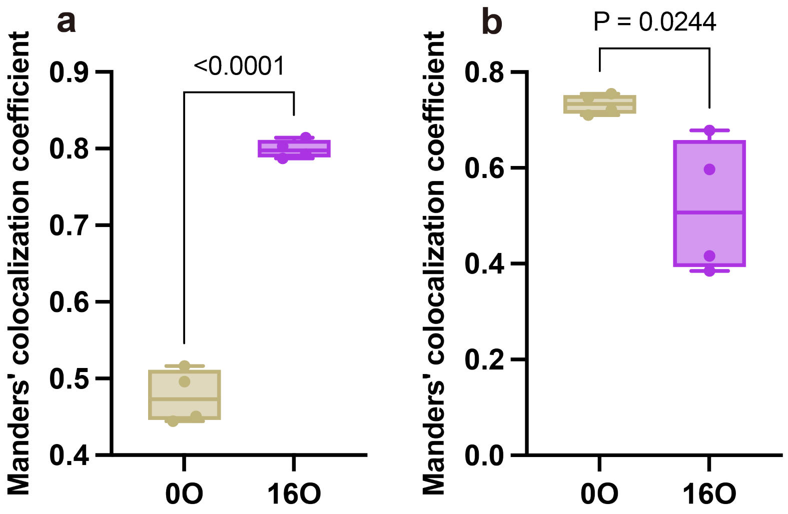

Supplementary Fig. 7 Manders' colocalization coefficient of nano emulsion with ER or lysosome. **a**, Manders' colocalization coefficient of 0O and 16O with the ERin DC2.4 cells. **b**, Manders' colocalization coefficient of 0O and 16O with the lysosome in DC2.4 cells. n=4.

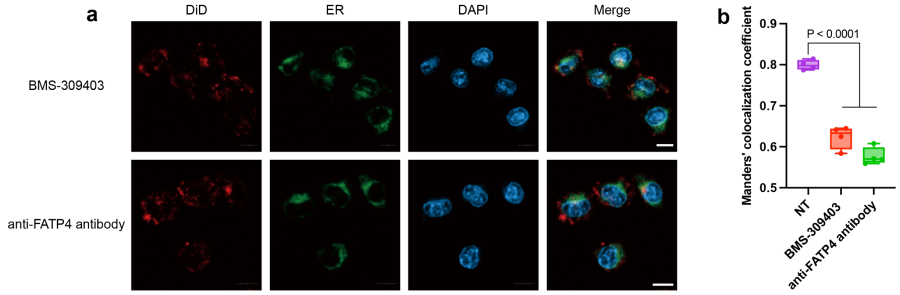

Supplementary Fig. 8 The ER colocalization of 16O with inhibition of FATP and FABP. Cells were pretreated with specific inhibitors (BMS-309403) or blocking antibodies (anti-FATP4 antibody) against FATP and FABP prior to incubation with 16O. **a**, ER colocalization was assessed by fluorescence imaging. **b**, The Manders' coefficients of ER and 16O.

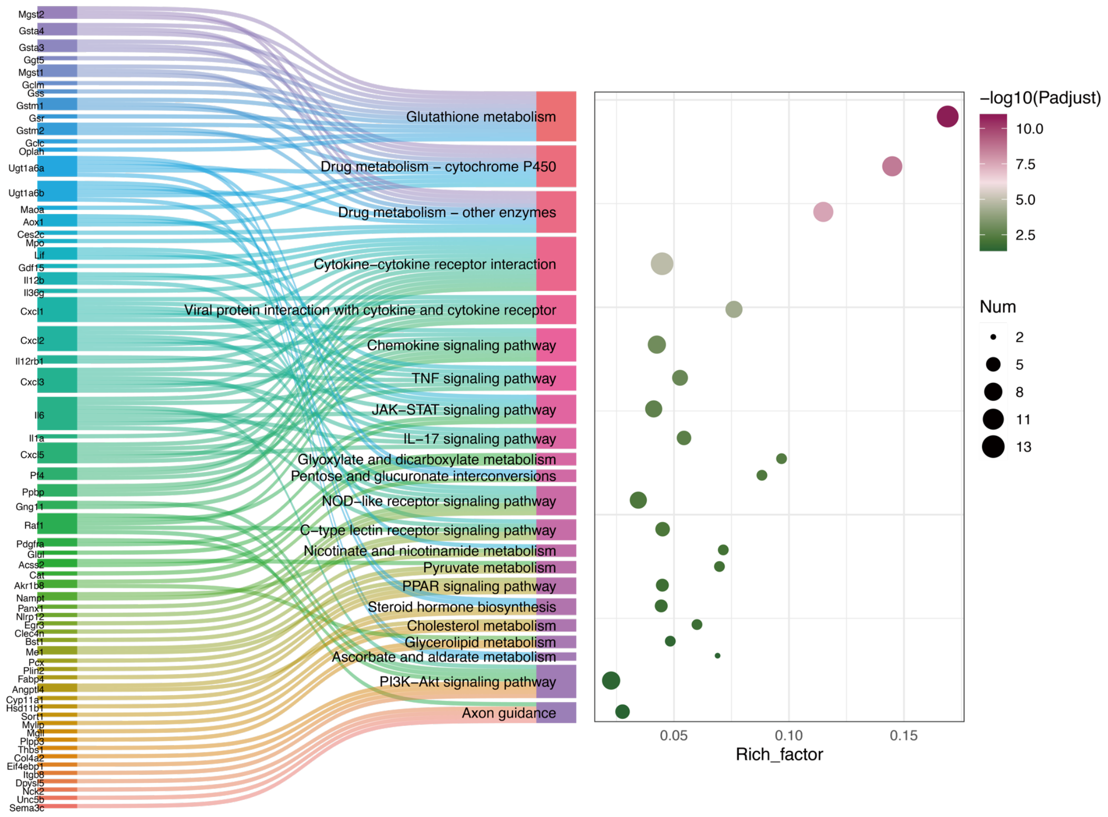

Supplementary Fig. 9 Compared with the control group PBS, KEGG enrichment analysis of up-regulated genes in BMDCs treated with 16O was performed by Mulberry + bubble plot (n = 3 per group).

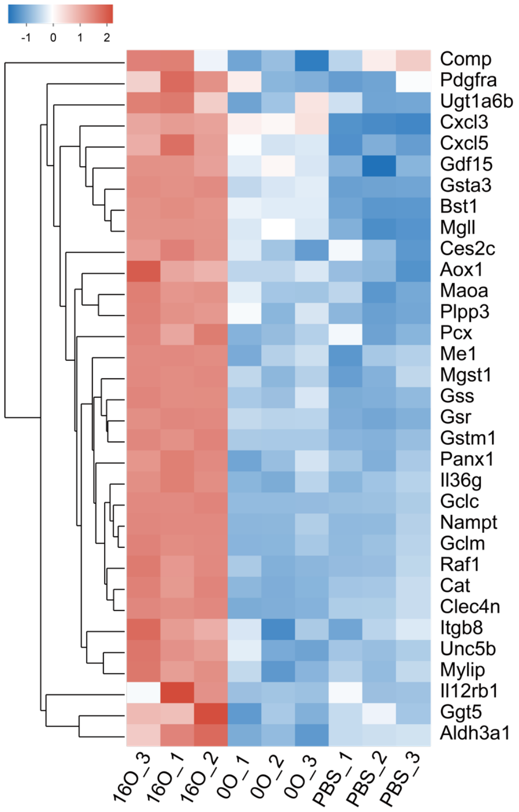

Supplementary Fig. 10 Heatmap showing clustered differentially expressed genes among PBS-, 0O-, and 16O-treated groups, highlighting distinct transcriptional profiles induced by 16O treatment. (n = 3 per group).

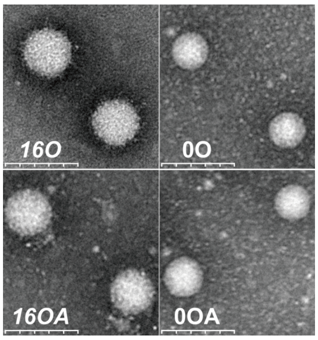

Supplementary Fig. 11 Transmission electron microscopy (TEM) images of the nanoemulsions. scale bar= 100 nm

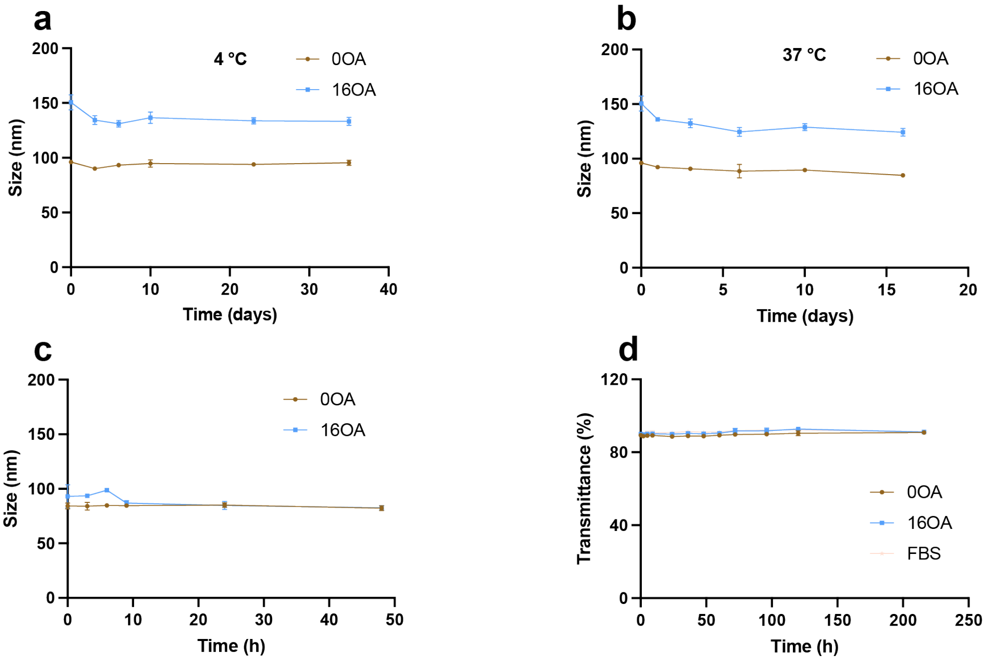

Supplementary Fig. 12 Characterization for the stability of preparation. **a**, The particle size curve of preparation stored under 4 °C (n = 3 per group). **b**, The particle size curve of preparation stored under 37 °C (n = 3 per group). **c**, The particle size curve of preparation co-incubated with 10% FBS under 37 °C (n = 3 per group). **d**, The transmittance curve of preparation co-incubated with 50% FBS under 37 °C (n = 5 per group).

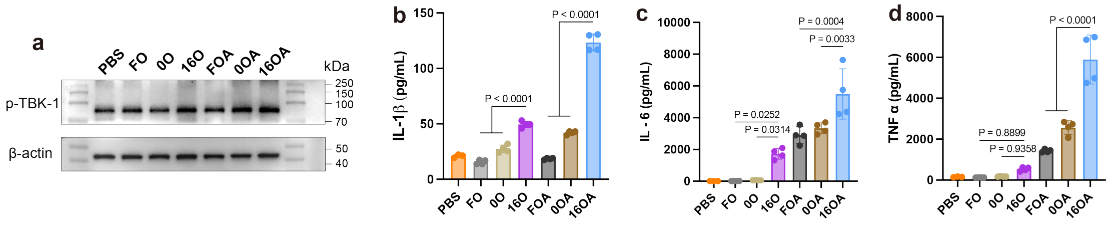

Supplementary Fig. 13 The activation of STING pathway. **a**, Phosphorylated TBK-1 expression of BMDCs. **b-d**, IL-1β, IL-6 and TNF-α secretion by BMDCs after treated with indicated formulations. (n = 4 per group).

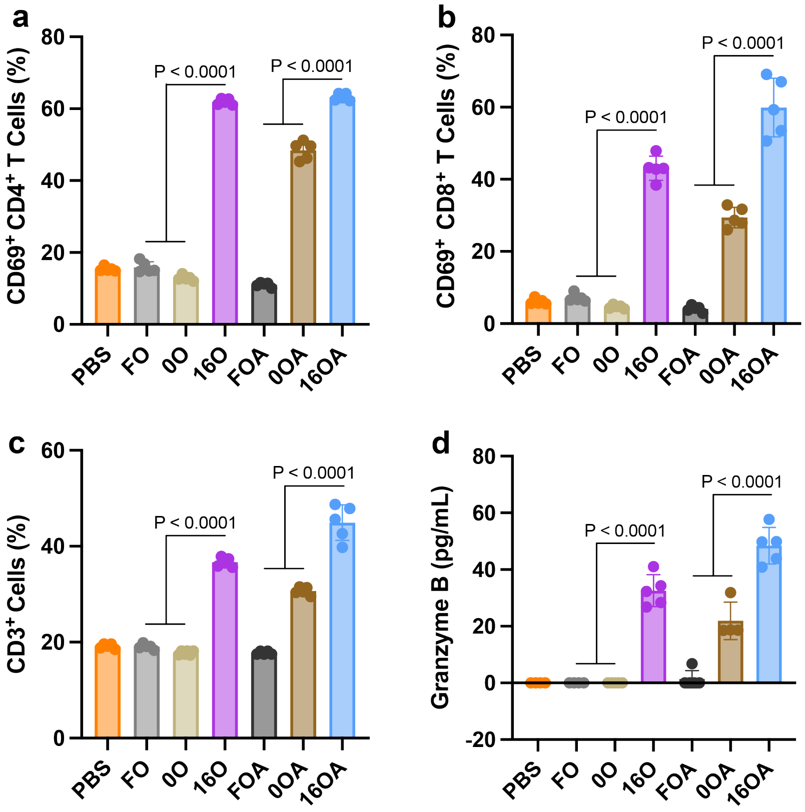

Supplementary Fig. 14 T cell activation and proliferation in vitro detection. **a**, CD4+T cell activation; **b**, CD8+T cell activation; **c**, T cell proliferation in vitro; **d**, Granzyme B secretion level in vitro. (n = 5 per group).

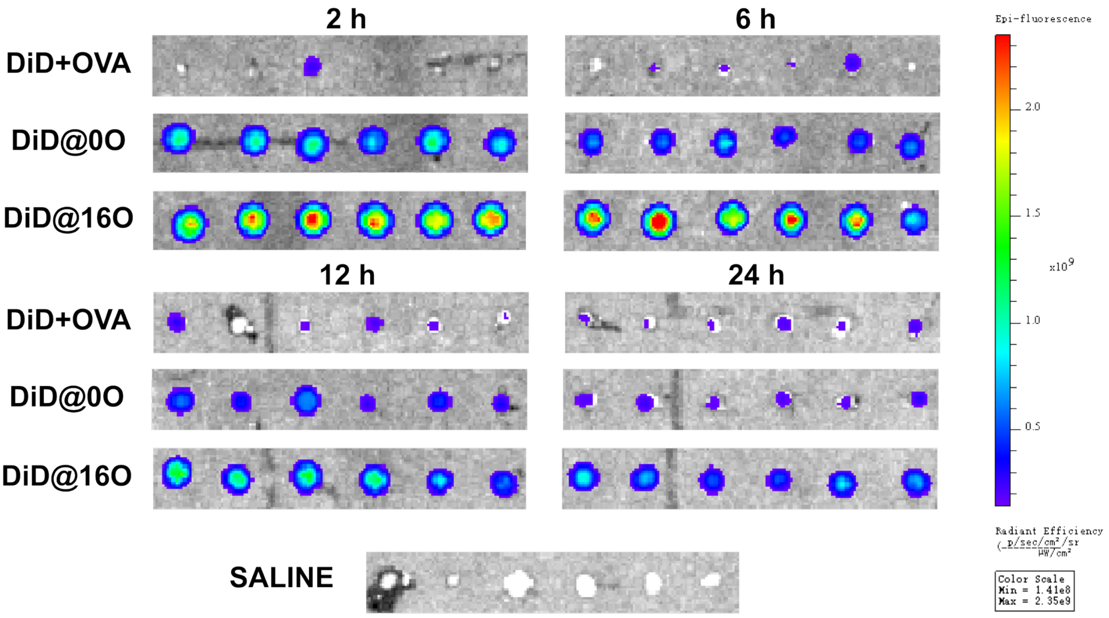

Supplementary Fig. 15 The DiD accumulation in popliteal lymph nodes at 2, 6, 12, and 24 h after subcutaneous injection of preparations containing fluorescent dye DiD on mice (n = 6 per group).

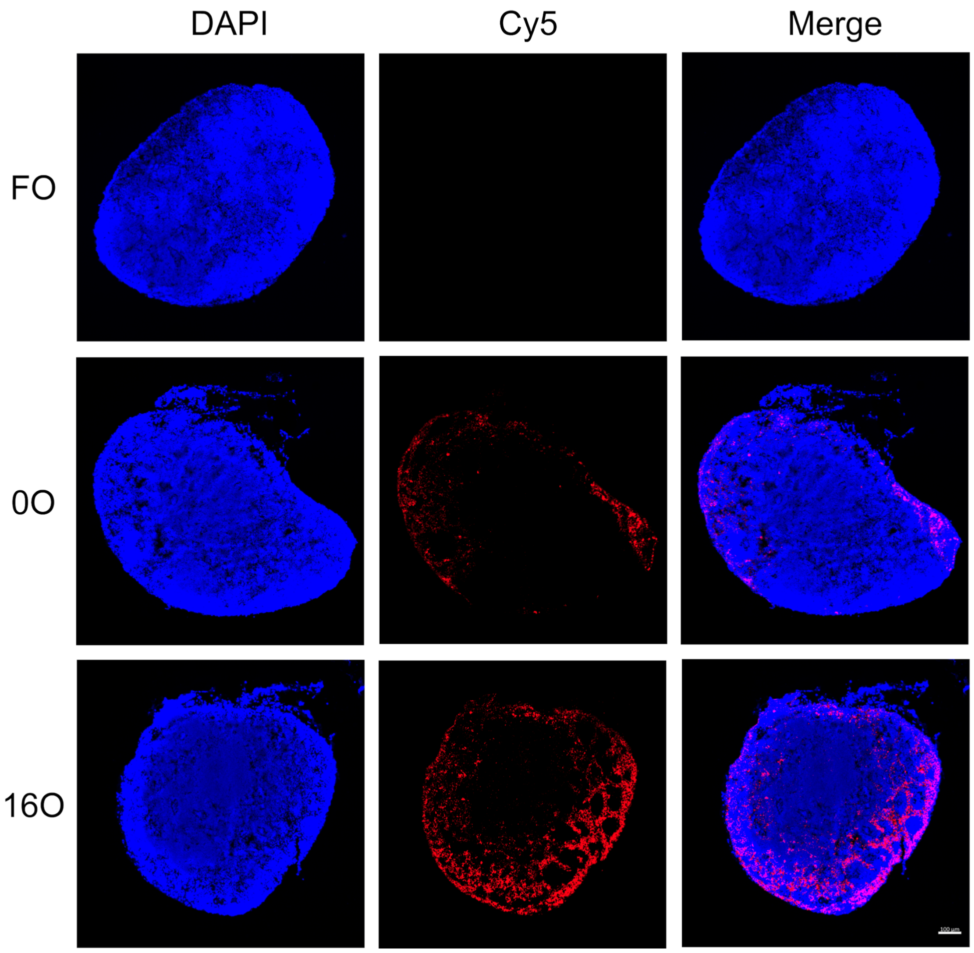

Supplementary Fig. 16 The OVA-CY5 distribution in popliteal lymph nodes section at 6 h after subcutaneous injection of preparations containing OVA-CY5 on mice, scale bar=100 μm.

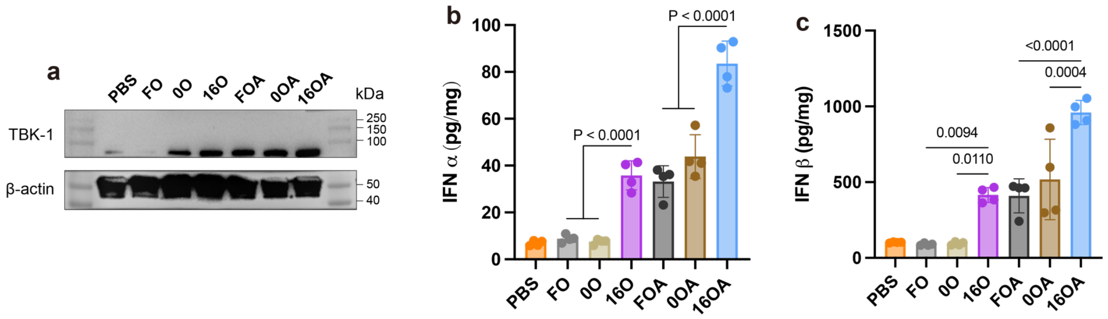

Supplementary Fig. 17 STING pathway activation in vivo. **a**, Western blot analysis of p-TBK1 expression levels in lymph nodes. **b-c**, ELISA quantification of IFN-α and IFN-β secretion in lymph nodes. (n=4 per group)

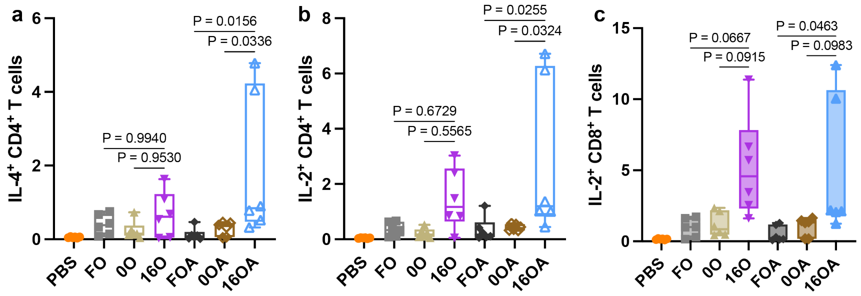

Supplementary Fig. 18 ICS results in splenic T lymphocytes from immunized mice (n = 6 per group): the level of $CD4^+$ cells expressing IL-4, IL-2, or $CD8^+$ T cells expressing IL-2.

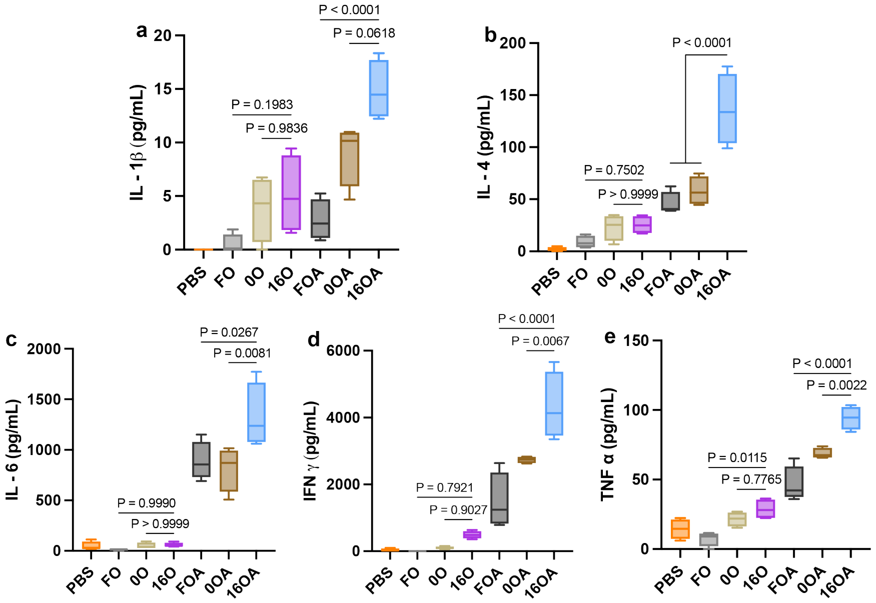

Supplementary Fig. 19 Spleen lymphocyte secretion levels of IL-1β (**a**), IL-4 (**b**), IL-6 (**c**), IFN-γ (**d**), TNF-α (**e**) (n = 4 per group).

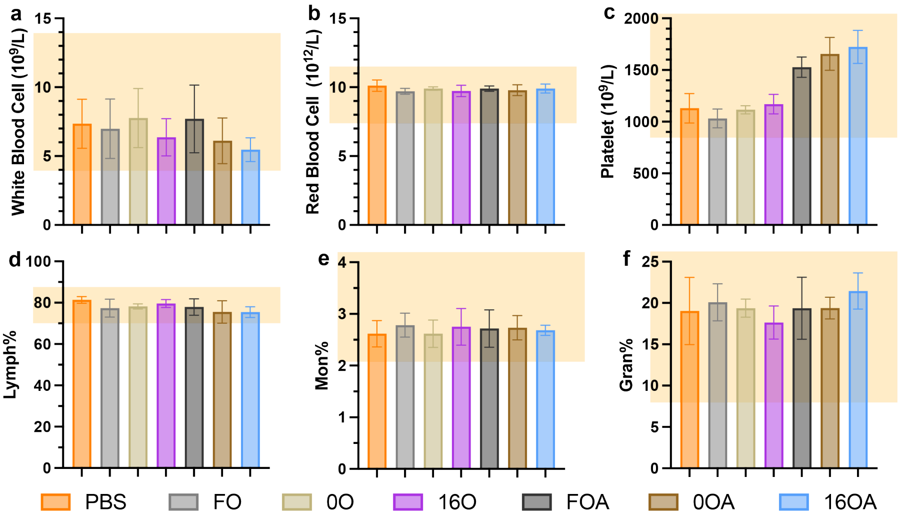

Supplementary Fig. 20 Complete blood count (CBC) analysis in immunized mice (n = 6): white blood cells (WBC) (**a**), red blood cells (RBC) (**b**), platelets (PLT) (**c**), lymphocytes (**d**), monocytes(**e**), and granulocytes (**f**). The shaded yellow area represents the normal reference range for female C57BL/6 mice (n = 6 per group).

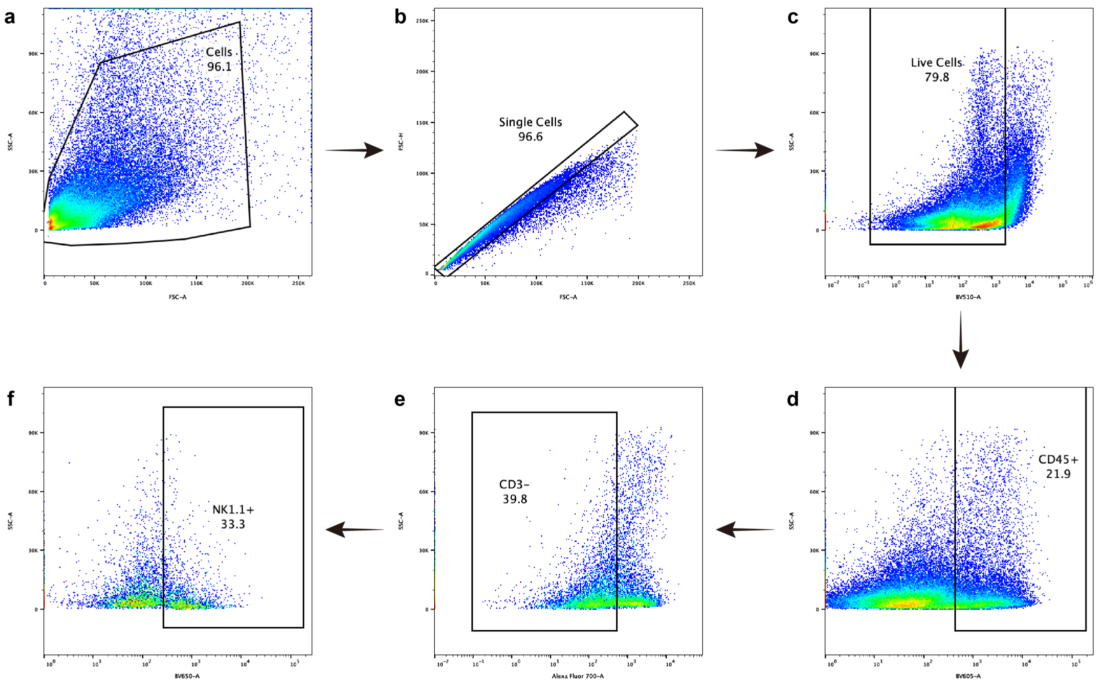

Supplementary Fig. 21 Representative gating strategy to identify NK cells within tumor microenvironment.

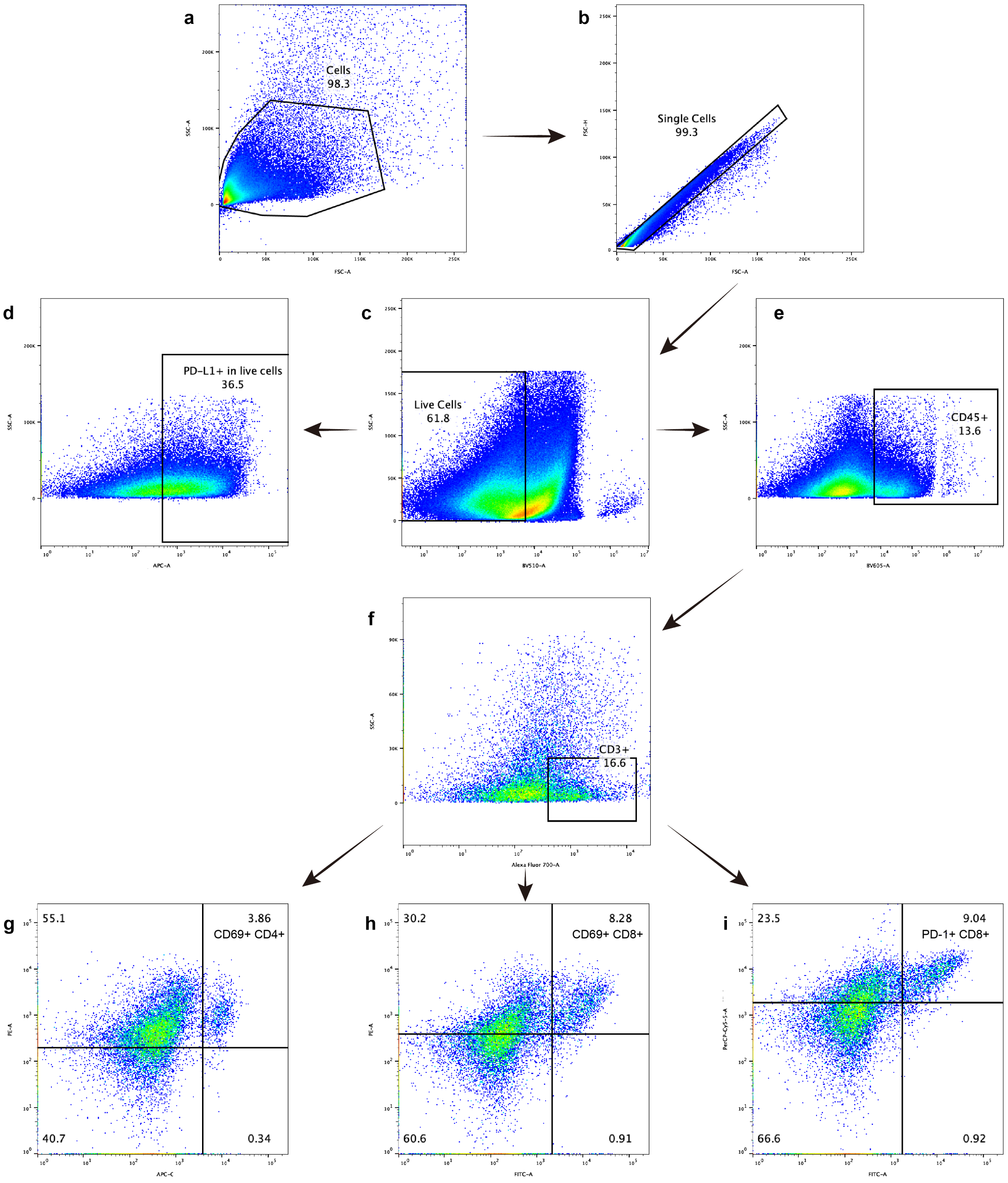

Supplementary Fig. 22 Representative gating strategy to identify expressing programmed death-ligand 1 (PD-L1) cells, $CD4^+$ cells expressing CD69, or $CD8^+$ T cells expressing CD69, programmed cell death protein-1 (PD-1) within tumor microenvironment.

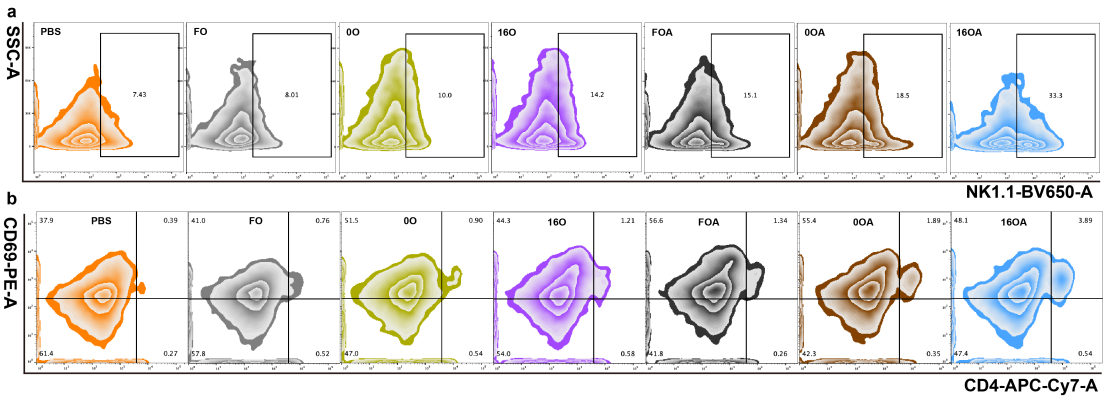

Supplementary Fig. 23 Representative flow cytometry plots showing the proportion of NK cells (**a**) and the activation levels of CD4$^+$ T cells (**b**).

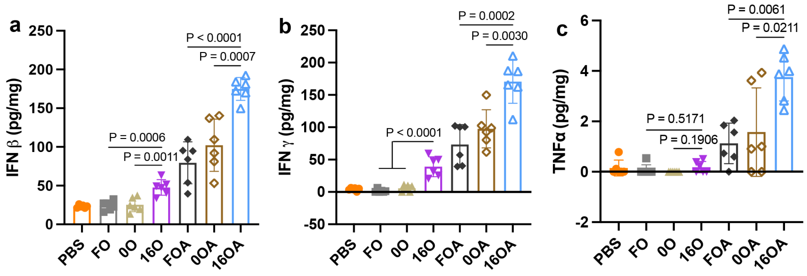

Supplementary Fig. 24 The expression levels of cytokines IFN-β, IFN-γ, and TNF-α within tumor microenvironment (n = 6 per group).

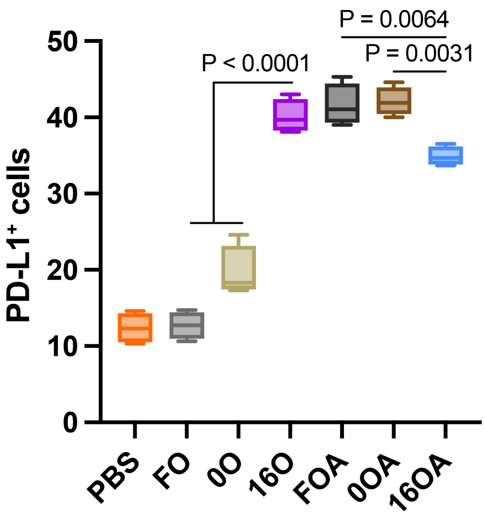

Supplementary Fig. 25 The proportion of cells expressing PD-L1 within tumor microenvironment (n = 4 per group).

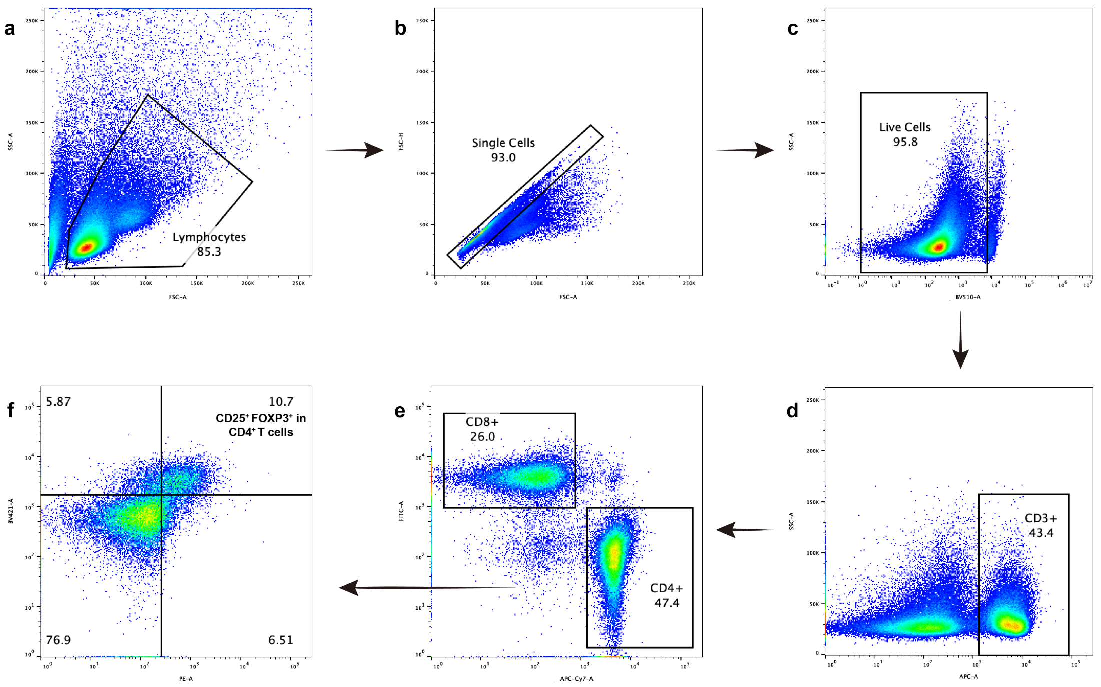

Supplementary Fig. 26 Representative gating strategy to identify Regulatory T cell (Treg) within the lymph nodes of tumor-bearing mice.

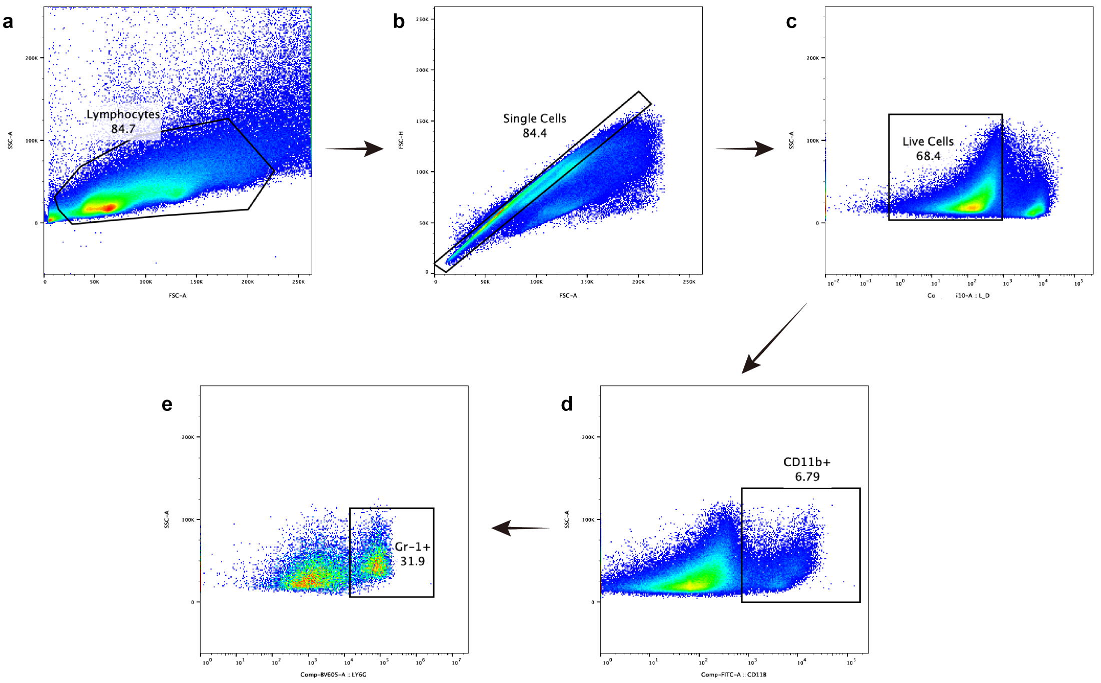

Supplementary Fig. 27 Representative gating strategy to identify myeloid-derived suppressor cells (MDSCs) within the spleens of tumor-bearing mice.

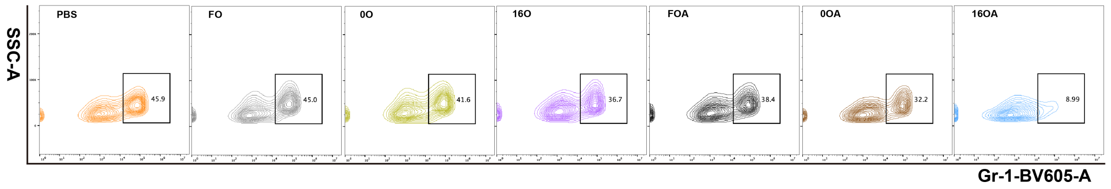

Supplementary Fig. 28 Representative flow cytometry plots showing the proportion of MDSCs within the spleens of tumor-bearing mice.

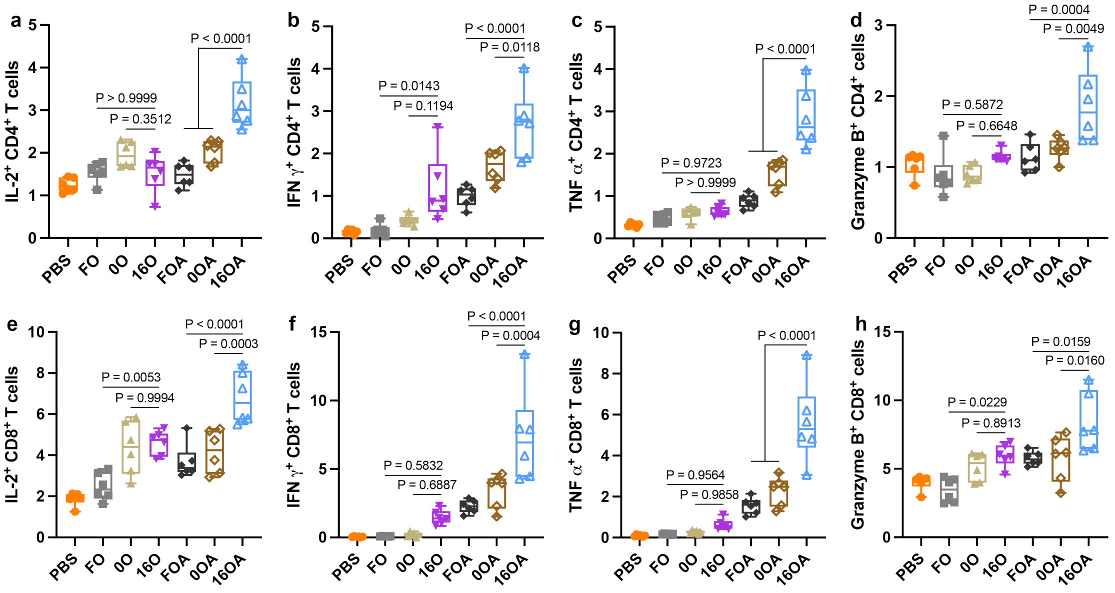

Supplementary Fig. 29 The expression levels of IL-2, IFN-γ, TNF-α, and Granzyme B in CD4⁺ and CD8⁺ T cells isolated from the spleens of tumor-bearing mice (n = 6 per group).

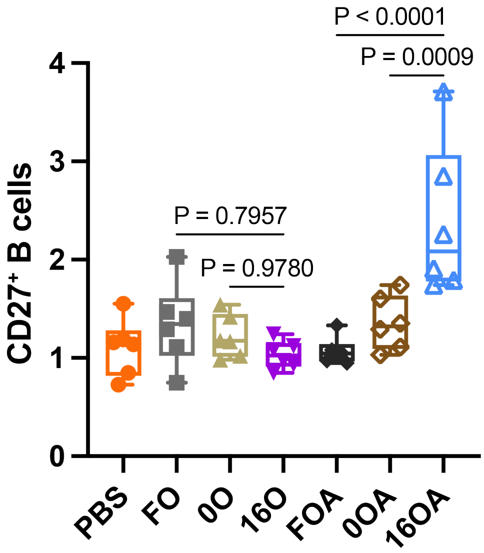

Supplementary Fig. 30 Memory B cell levels in the spleen of tumor-bearing mice treated with different preparations (n = 6 per group).

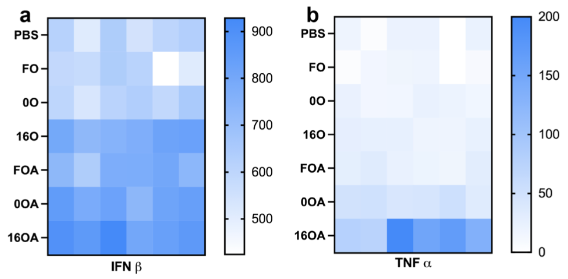

Supplementary Fig. 31 The secretion levels of IFN-β (**a**) and TNF-α (**b**) by splenic lymphocytes of tumor-bearing mice (n = 6 per group).

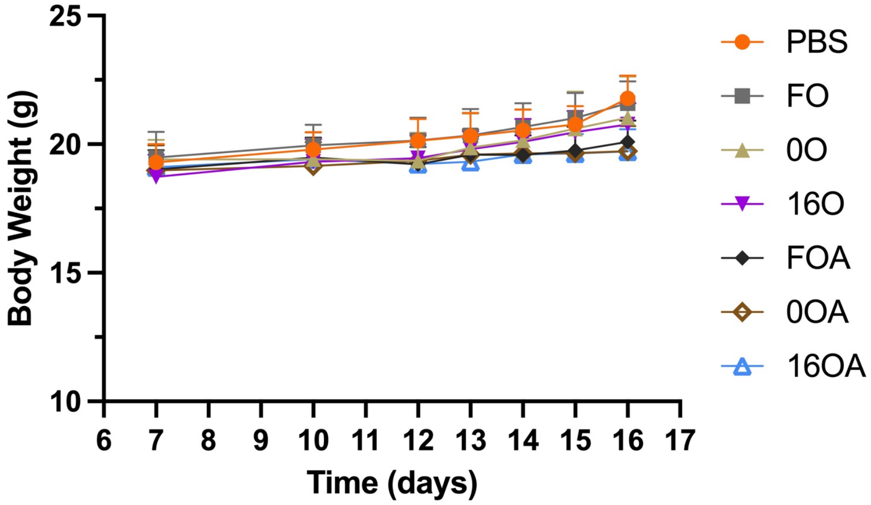

Supplementary Fig. 32 Weight curve of tumor-bearing mice during different preparations treatment (n = 9 per group).

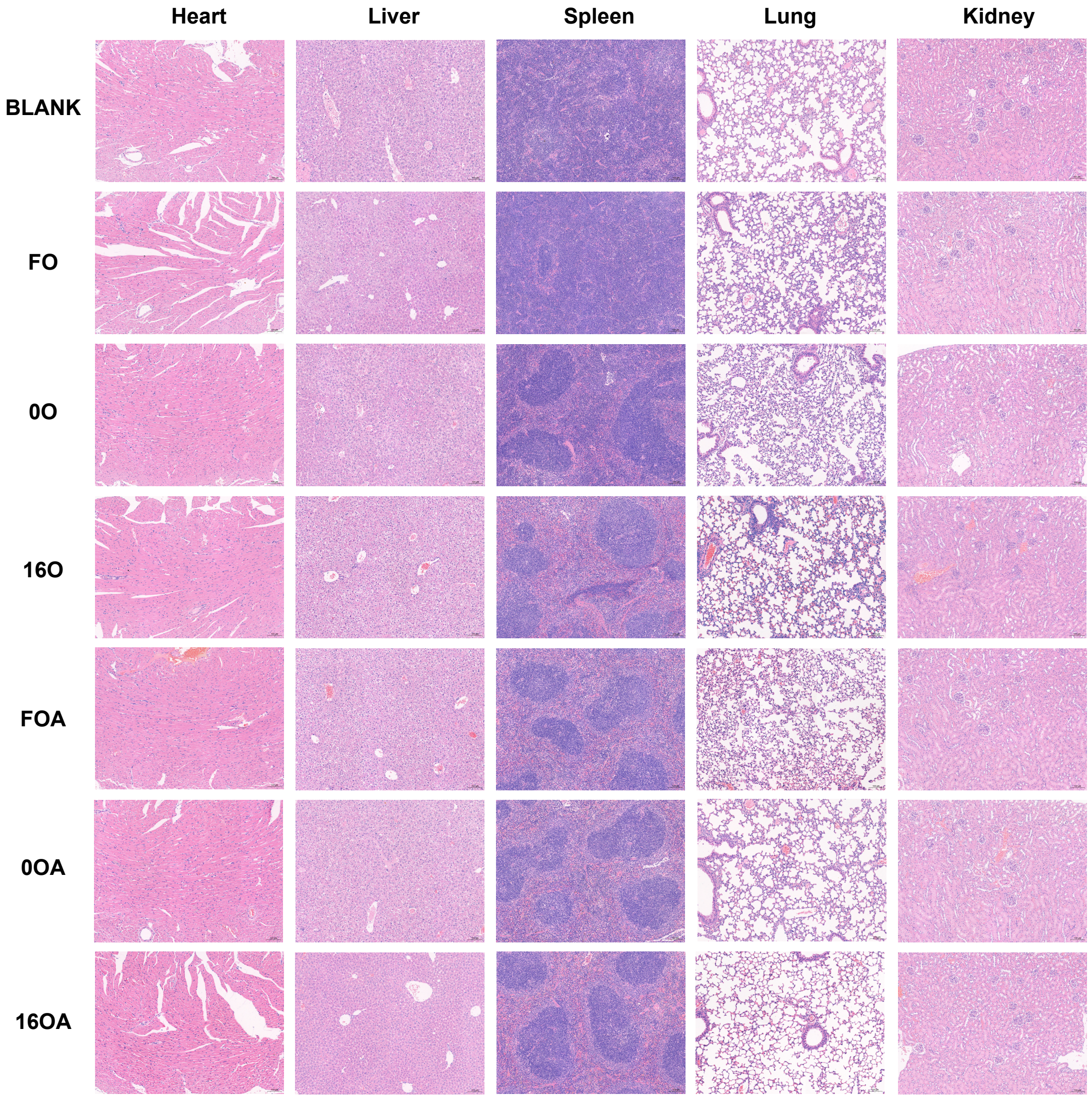

Supplementary Fig. 33 HE stained sections of heart, liver, spleen, lung and kidney from tumor-bearing mice after different preparations treatment.

Supplementary tables

Table S1. The material characterization for a series of fatty acid-modified and unmodified HSA.

	Substitution degree (Mean ± SD)	Molecular weight (kDa)	Isoelectric point
0	--	66.40	4.47-4.53
2	12.37 ± 0.40	66.71	3.87-3.95
6	14.93 ± 0.45	67.26	4.17-4.22
8	14.07 ± 0.14	67.45	4.22-4.30
10	13.01 ± 0.55	67.58	4.22-4.30
12	14.57 ± 0.12	67.97	4.38-4.47
14	18.74 ± 0.05	68.73	4.38-4.47
16	19.72 ± 0.14	69.17	4.22-4.30
18	19.51 ± 0.37	69.47	4.22-4.30
Ole	20.45 ± 0.36	69.59	4.38-4.47

Table S2. The preparation characterization for nano-emulsions loaded with OVA (n = 3 per group).

	Size (nm)	PDI	Zeta Potential (mV)	EE of OVA (%)
0O	94.04 ± 1.45	0.239 ± 0.028	-17.17 ± 0.76	98.2 ± 0.7
2O	90.61 ±1.69	0.190 ± 0.032	-25.97 ± 1.53	96.6 ± 2.5
6O	92.39 ± 1.03	0.195 ± 0.029	-25.97 ± 0.93	98.0 ± 1.0
8O	144.07 ± 2.12	0.220 ± 0.041	-25.53 ± 0.49	97.0 ± 1.3
10O	155.47 ± 1.54	0.195 ± 0.047	-22.37± 0.90	96.7 ± 2.3
12O	151.33 ± 2.22	0.235 ± 0.026	-29.37 ± 1.31	96.7 ± 2.4
14O	151.50 ± 1.45	0.219 ± 0.042	-28.3 ± 0.98	95.9 ± 1.4
16O	146.40 ± 3.30	0.218 ± 0.016	-23.87 ± 0.46	97.2 ± 1.8
18O	110.47 ± 2.49	0.231 ± 0.008	-25.23 ± 0.45	96.8 ± 1.4
OleO	160.40 ± 6.97	0.245 ± 0.035	-30.8 ± 1.47	95.7 ± 0.5

Table S3. The preparation characterization for nano-emulsions loaded with OVA and diABZI (n = 3 per group).

	Size (nm)	PDI	Zeta Potential (mV)	EE of OVA (%)	EE of diABZI (%)
0OA	96.11 ± 0.93	0.239 ± 0.021	-12.90 ± 0.56	95.8 ± 1.7	97.8 ± 1.4
16OA	150.53 ± 6.84	0.207 ± 0.038	-23.53 ± 0.47	97.9 ± 1.5	99.1 ± 0.9

Table S4. Antibodies used for flow cytometry in the study.

Antibodies	Company	Catalog #	Dilution
Fixable Viability Dye eFluor™ 506	Invitrogen	65-0866-18	1:1000
Fc Block™	BD	553142	1:50
BB700 Anti-CD11c	BD	566504	1:100
BV650 Anti-CD40	BD	740492	1:100
FITC Anti-CD80	Invitrogen	11-0801-82	1:200
APC Anti-CD86	Invitrogen	17-0862-82	1:330
FITC Anti-CD8a	BD	553030	1:100
FITC Anti-CD11b	BD	557396	1:100
PE Anti-F4/80	BD	565410	1:100
PE Anti-CD19	Invitrogen	12-0193-82	1:160
BV421 Anti-Ly6G	BD	562737	1:100
Alexa Fluor 700 Anti-CD3e	BD	557984	1:100
PE Anti-H2Kb-SIINFEKL	Invitrogen	14-5743-81	1:200
APC-eFluor 780 Anti-CD4	Invitrogen	47-0041-82	1:160
PE Anti-CD69	Invitrogen	12-0691-82	1:80
APC Anti-B220	Invitrogen	17-0452-82	1:80
Alexa Fluor 488 Anti-GL7	Invitrogen	53-5902-82	1:100
PE-CY7 Anti-CD27	Invitrogen	25-0271-82	1:40
PerCP Cyanine5.5 Anti-IL-2	Invitrogen	45-7021-82	1:160
PE Anti-IL-4	Invitrogen	12-7041-81	1:160
APC Anti-IFN-γ	Invitrogen	17-7311-81	1:160
eFlour 450 Anti-TNF-α	Invitrogen	48-7321-82	1:80
PE Anti-CD62L	Invitrogen	12-0621-82	1:160
APC Anti-CD44	Invitrogen	17-0441-82	1:330
PE Anti-CD25	BD	553075	1:100
BV421 Anti-FOXP3	BD	562996	1:100
BB700 Anti-PD-1	BD	566514	1:100
APC Anti-PD-L1	BD	564715	1:100
BV650 Anti-NK-1.1	BD	564143	1:100
BV605 Anti-CD45	BD	563053	1:100
PerCP-eFluor 710 Anti-CD206	Invitrogen	46-2061-82	1:160
APC-CY7 Anti-Gr-1	Invitrogen	47-5931-82	1:40
BV786 Anti-CD1d	BD	740902	1:100

FITC Anti-CD5	BD	553020	1:100
BV421 Anti-CD21	BD	562756	1:100
APC Anti-IL-10	BD	554468	1:100
PE Anti-Granzyme B	Invitrogen	12-8898-82	1:160
APC Anti-CD3	Invitrogen	17-0032-82	1:40
BB700 Anti-CD69	BD	566500	1:100

Table S5. The uptake inhibitors or conditions.

Inhibitors/ conditions	Concentrations
4℃	-
Nystatin	25 μM
poly-L-lysine	200 μg/mL
Dextran sulfate sodium (DSS)	100 μg/mL
Chlorpromazine	20 μM
Methyl-β- cyclodextran	10 μM
Amiloride	100 μM
sucrose	154 mg/mL
